# Supplementary material for: Complementary resource preferences spontaneously emerge in diauxic microbial communities
Source: Nat Commun. 2021 Nov 18;12:6661. doi: 10.1038/s41467-021-27023-y (PMC8602314; doi:10.1038/s41467-021-27023-y)
Supplement: Supplementary file 1 — Supplementary Information [file 41467_2021_27023_MOESM1_ESM.pdf]

Supplementary Information
Complementary resource preferences spontaneously emerge in diauxic microbial communities

Zihan Wang[1,2], Akshit Goyal[3], Veronika Dubinkina[2,4],
Ashish B. George[2,5], Tong Wang[1,2], Yulia Fridman[6], and Sergei Maslov[1,2,4*]

[1] *Department of Physics, University of Illinois at Urbana-Champaign, Urbana, IL 61801, USA.*
[2] *Carl R. Woese Institute for Genomic Biology, University of Illinois at Urbana-Champaign, Urbana, IL 61801, USA.*
[3] *Physics of Living Systems, Department of Physics, Massachusetts Institute of Technology, Cambridge, MA 02139, USA.*
[4] *Department of Bioengineering, University of Illinois at Urbana-Champaign, Urbana, IL 61801, USA.*
[5] *Department of Plant Biology, University of Illinois at Urbana-Champaign, Urbana, IL 61801, USA.*
[6] *National Research Center "Kurchatov Institute", Moscow, 123182, Russia*

This file contains:

Supplementary Figures, Fig. S1 to S6
Supplementary Text, sections A to I

Supplementary Figures

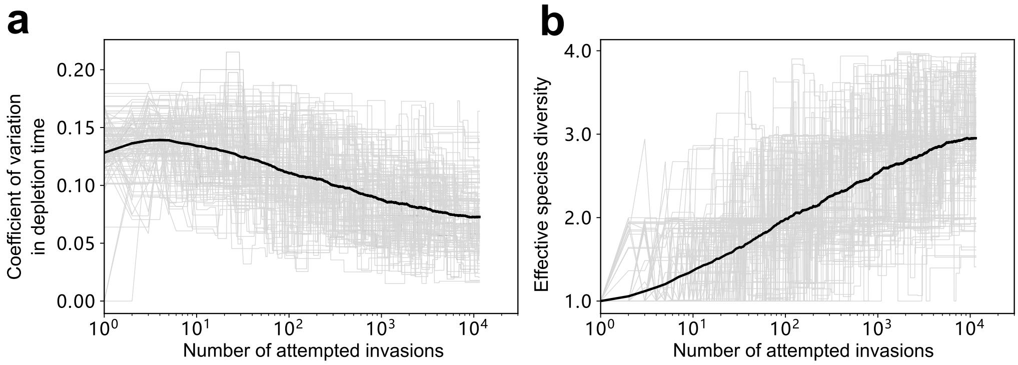

Supplementary Figure 1: **Community-level properties during the assembly process.** Data from the same set of simulations as in Fig. 2. Solid lines in all subplots are averaged over all simulated communities at each invasion during the assembly process. Grey lines correspond to 100 random individual community assembly simulations. **(a)** Coefficient of variation in T_i's slowly decreases during the assembly process. **(b)** Effective diversity, here defined by inverse Simpson's index, increases during the assembly.

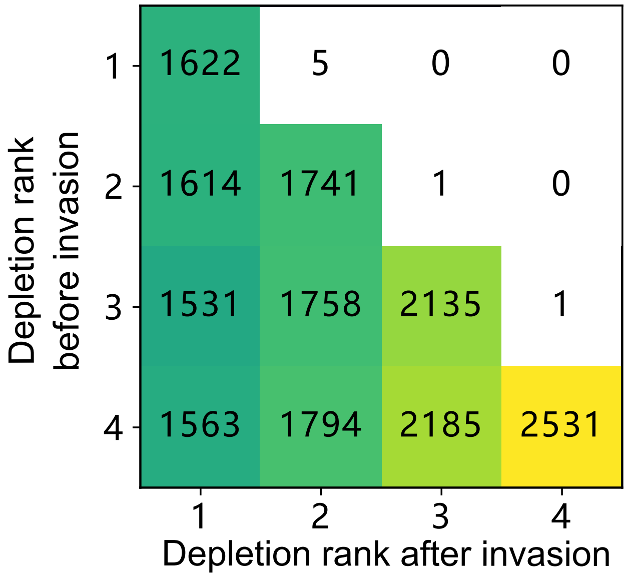

Supplementary Figure 2: **Depletion order of the invader's top choice before and after the invasion.** Data from the same set of simulations as in Fig. 2. To eliminate the unnecessary randomness in the early stage of assembly, successful invasions before the 100th attempt were not counted. In each grid there displays the count of the corresponding event.

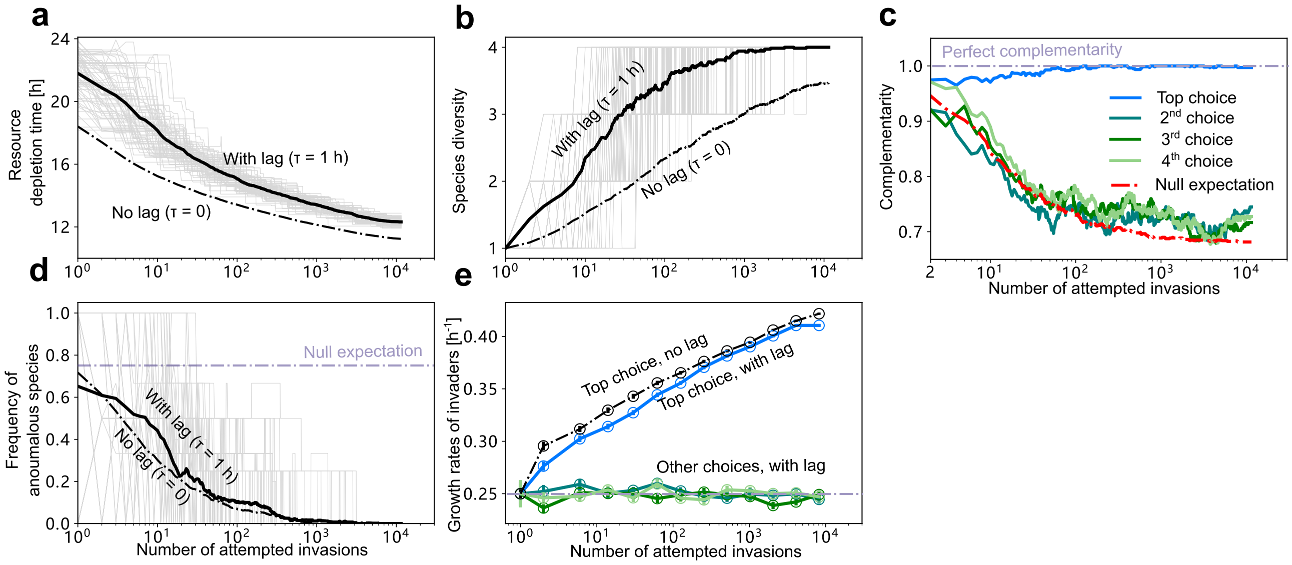

Supplementary Figure 3: **Effects of a finite lag on community assembly** Both "with lag" and"no lag" data come from the same set of simulations as in Fig. 5. In (a), (b) and (d) the grey lines display the data from the 100 independent community assembly simulations where the lag time is 1 hr. In (e), each quantity represents a moving average from these 100 simulations. Error bars represent s.e.m.

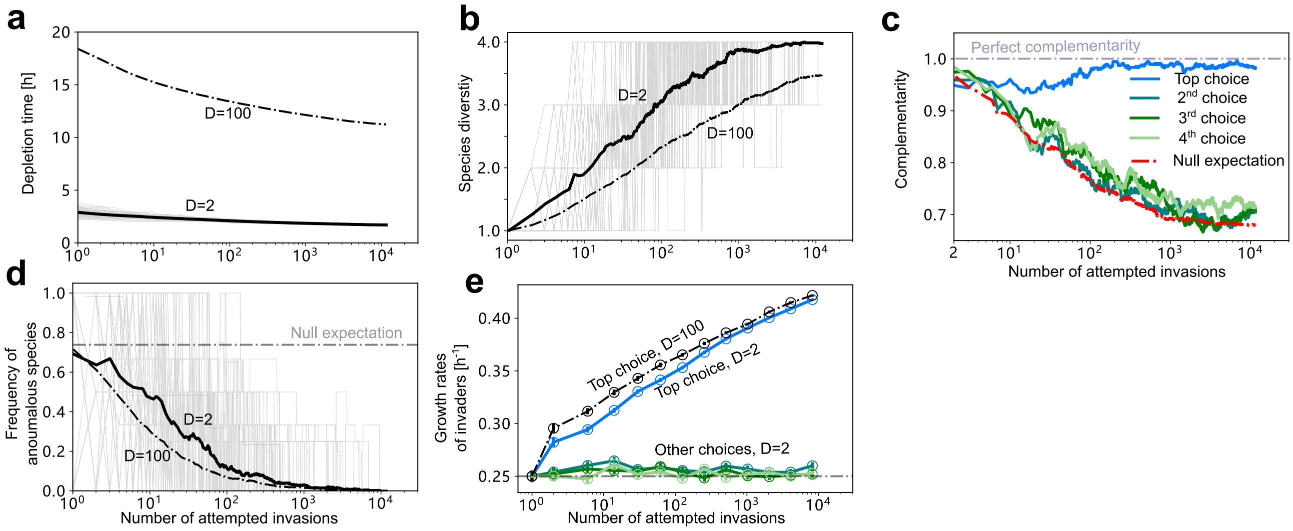

Supplementary Figure 4: **Effects of dilution factor being small on community assembly** Data of $D = 100$ comes from the same set of simulations as in Fig. 2. The dilution factor D is the only modified parameter when simulating the $D = 2$ data. In (a), (b) and (d) the grey lines display the $D = 2$ data, which contains 100 simulations of independent community assembly. In (e), each quantity represents a moving average from these 100 simulations. Error bars represent s.e.m.

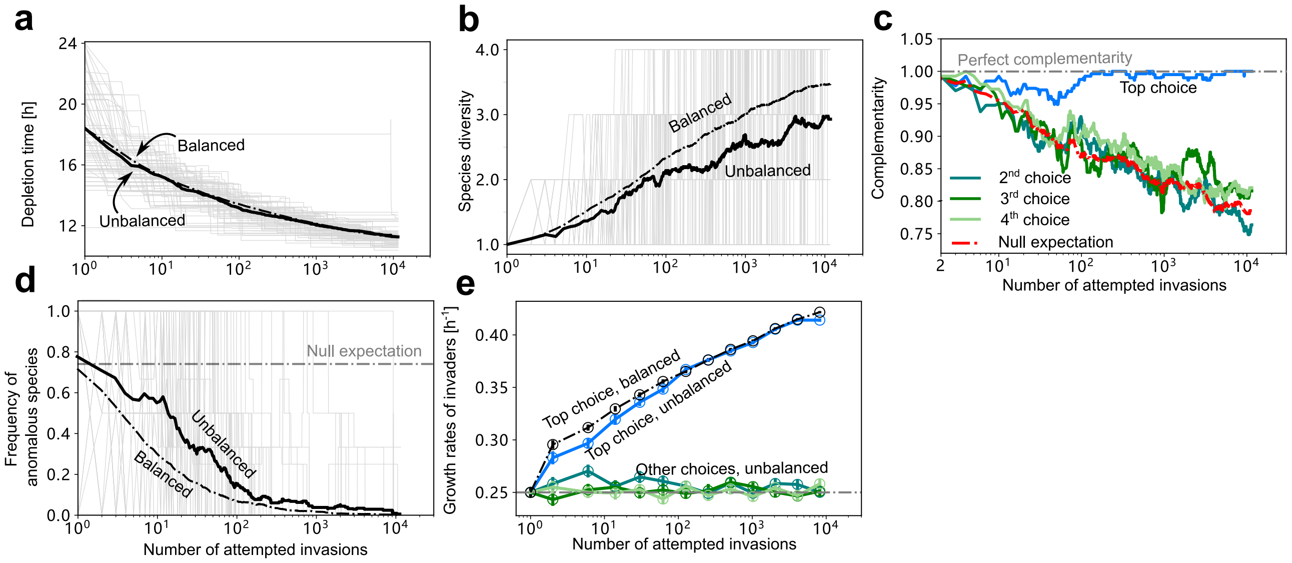

Supplementary Figure 5: **Effects of extremely unbalanced nutrient supply on community assembly** Data of balanced resources comes from the same set of simulations as in Fig. 2. As for the unbalanced resources, the 4 resources were set to a ratio of 100:1:1:1 at the beginning of each dilution cycle, with the sum of all resource concentrations kept at the same level as in the balanced case. In (a), (b), and (d) the grey lines display the data of unbalanced resources, which contains 100 simulations of independent community assembly. In (e), each quantity represents a moving average from these 100 simulations. Error bars represent s.e.m.

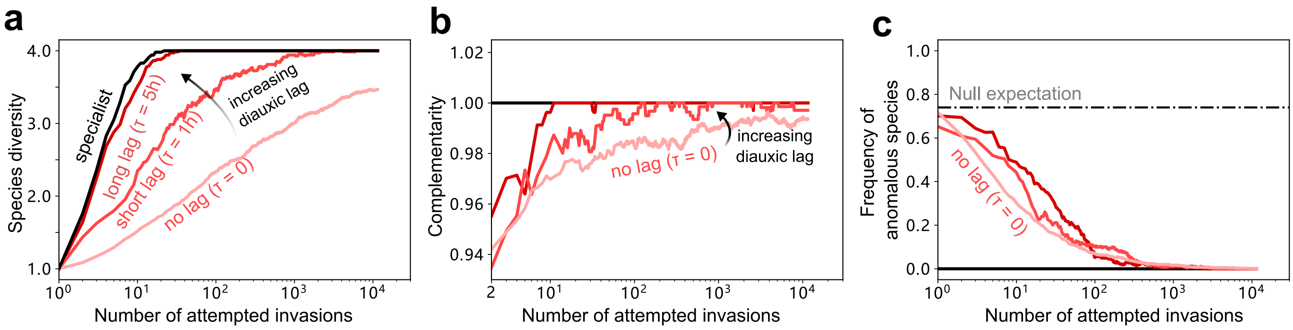

Supplementary Figure 6: **Compare diauxic shifters with lags to specialists** All diauxic shifters' data come from the same set of simulations as in Fig. 5. The data of the specialists were simulated in the following manner. We generated the top choice growth rates of those specialists from the same distribution ($N(0.5, 0.1^2)$ rectified at 0.1). We then introduced these species one by one by the following rule: after each invasion, on each resource, the specialists with the highest growth rates win and stay. This includes 100 independent community assembly processes. In all three panels, the black lines correspond to the specialists, the red lines display the data from lag time 5 hr, the orange lines show the case of lag time 1 hr, and the brown lines correspond to simulations without lag.

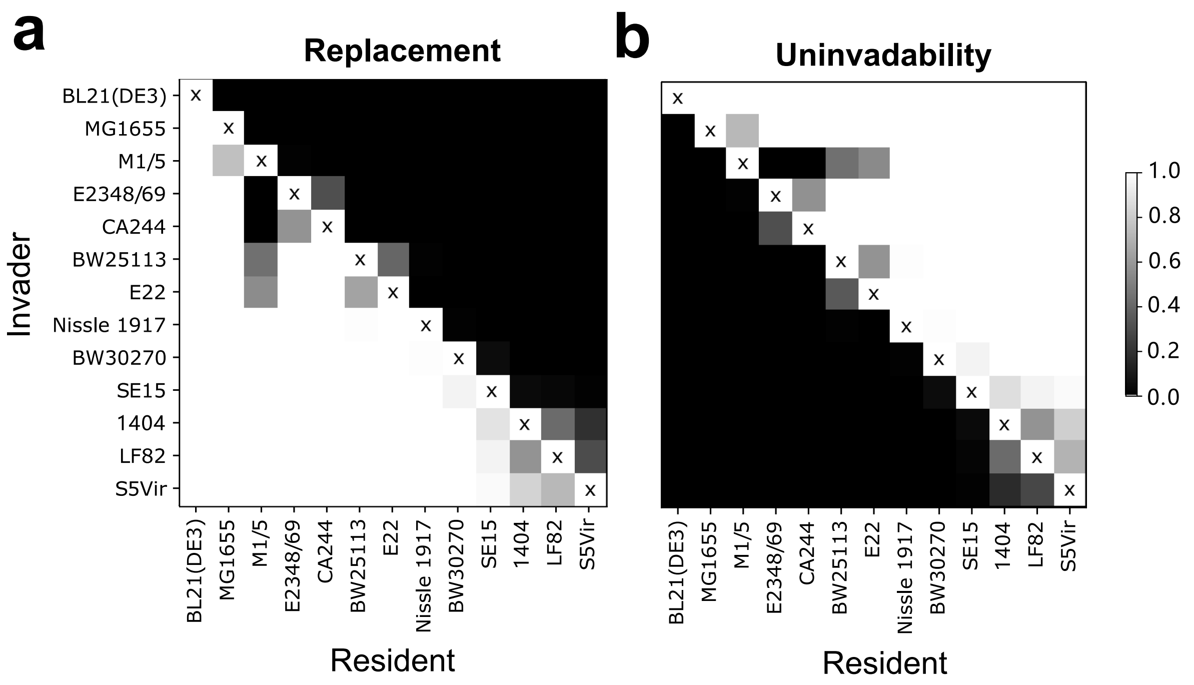

Supplementary Figure 7: **Numerical invasion experiments of *E. coli* strains studied in [1].** A strain on the y-axis invades the monoculture of a strain on the x-axis, and the greyscale represents the frequency of both strains survive in the steady state, counted from 100 runs where lag time values were individually sampled. In **(a)** shows the frequency where the invader replaces the resident, and in **(b)** shows the frequency where the invader does not survive in the end.

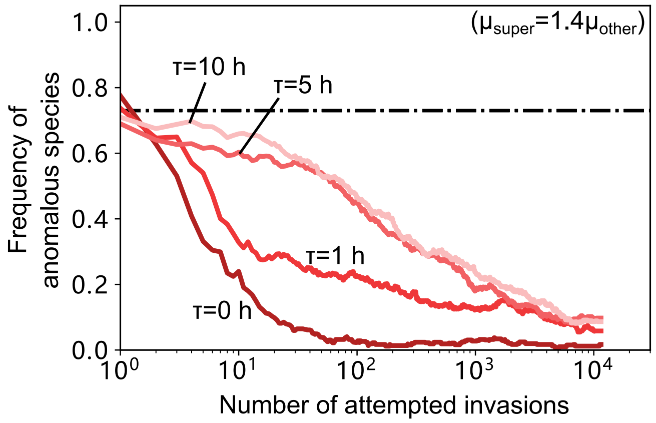

Supplementary Figure 8: **Effects of having unbalanced growth rate distributions on different resources** Growth rate distribution on a "super" resource R_{super} is set at $N(\mu_{super} = 0.35, 0.05^2)$, while such distribution on the rest 3 resources remain at $N(\mu_{other} = 0.25, 0.05^2)$. All other parameters are the same as the simulations regarding Fig. 2. Lags promote the survival of anomalous species when a "super" resource is present. The black dash-dotted line shows the proportion of anomalous species in the species pool. Lines of shades of red display frequency of anomalous species during the community assembly, averaged over 100 community assembly simulations.

Supplementary Text
Complementary resource preferences spontaneously emerge in diauxic microbial communities

Contents

A Depletion order and times in the light of our Tilman-inspired approach 7

B Pareto-optimal boundary and the one-microbe line 13
B.1 Relation to Tilman's graphical method 13
B.2 The Pareto-optimal boundary 13
B.3 One-microbe line 14
B.4 Types of the steady state coexistence: stable and unstable 16

C Anomalous microbes and their rarity 17

D Multistability in a community of diauxic shifters 20
D.1 What prompts multistability 20
D.2 An example of bistability between two single-microbe states 20
D.3 An example of bistability between coexistence and exclusion for two microbial species 21
D.4 Conclusions 21

E On the attainability of the final steady state: does the invasion process end? 22
E.1 An example of a cyclic final solutions 22
E.2 Why the invasion process ends 23

F On the number of surviving species 25
F.1 Steady state survivors 25
F.2 Why the competitive exclusion principle applies 25
F.3 Trade-off miraculous diversity boost takes too much to be manifested 25
F.4 Conclusion: the regular rules of the competitive exclusion apply 26

G How different yields affect the outcome of the serial dilution experiment 26

H Taking lags into account 28
H.1 Multistability induced by lags 32

I Representing the co-utilizing microbes in the time plane 35

J Coexistence of the diauxic species having the same preferential orders 37

K What happens to complementarity and diversity when the growth rate distributions are not similar for different sources 40
K.1 The criterion for the presence of the complementarity, lags neglected 41
K.2 When the diauxic lags enter the picture: complementarity preserved and the anomalous microbes getting their chance 43
K.3 In which way non-similarity of the growth rate distributions on different sources affects diversity 44
K.4 Multiple resources, one supernutrient 45

A Depletion order and times in the light of our Tilman-inspired approach

In a standard serial dilution experiment, resources get resupplied in the same fixed amounts at the beginning of each dilution cycle. The microbial community populating the environment comes to each new step diluted $D-$fold, compared to the values of the species concentrations acquired at the end of the previous dilution cycle. D here is the dilution coefficient.

Depletion order is the order in which resources disappear from the environment one by one during one given dilution cycle, getting consumed by the microbes.

Consider first a single microbe invading an abiotic medium containing two resources in the concentrations R_1 and R_2 (by R capital we designate both resources and their concentration values, depending on the context). Suppose R_1 precedes R_2 in the microbe's personal preferential list. If the bacterium invades in a seed amount, it may not be able to consume even R_1 during the first dilution cycle. If that is the case, during the first cycle it does not even touch R_2. However, if the cycle lasts long enough, the microbe grows more than $D-$fold during the step, so that it comes to the next dilution step in greater concentration. Eventually it gets fast enough to consume both R_1 and R_2, one by one in a diauxie manner, in the order determined by its own list of preferences. When it reaches the point of growing precisely $D-$fold during a dilution step, it comes to every next cycle with the same initial concentration as to the previous one, and thus, in a sense, a steady state arises. Let T_1 be the total lifetime of the resource R_1 and T_2 that of R_2 during a dilution cycle. Obviously, as R_1 gets consumed first, $T_2 > T_1$, and T_2 ends up to be the effective time of the microbe growth during the dilution cycle.

For the steady state, we write:

$$N_\alpha \exp\left(g_{\alpha 1} T_1 + g_{\alpha 2}\left(T_2 - T_1\right)\right) = D N_\alpha, \tag{S.1}$$

so that

$$g_{\alpha 1} T_1 + g_{\alpha 2}\left(T_2 - T_1\right) = \log\, D, \tag{S.2}$$

where N_α is the initial steady state concentration of the bacterial species B_α, $g_{\alpha i}$ is its growth rate on the resource number i, time T_1 the bacterium spends growing exponentially on the resource R_1 and for the rest $T_2 - T_1$ it grows on the resource R_2.

The steady state concentration N_α and the times T_1, T_2 are determined by the initial (for each cycle) resource concentrations R_1 and R_2 in the following way. First, starting a new dilution cycle with the initial steady state concentration N_α, the microbe ends it having consumed both R_1 and R_2, so that

$$D N_\alpha = N_\alpha + R_1 + R_2, \tag{S.3}$$

or else

$$N_\alpha = \frac{R_1 + R_2}{D - 1}, \tag{S.4}$$

note that in the above we do not take yields into account.

As the resource R_1 gets consumed in the time period T_1 (the depletion time of R_1)

$$N_\alpha\, \exp\left(g_{\alpha 1} T_1\right) = N_\alpha + R_1, \tag{S.5}$$

which, combined with $(S.4)$, gives

$$T_1 = \frac{1}{g_{\alpha 1}}\, \log\, \left(\frac{D R_1 + R_2}{R_1 + R_2}\right). \tag{S.6}$$

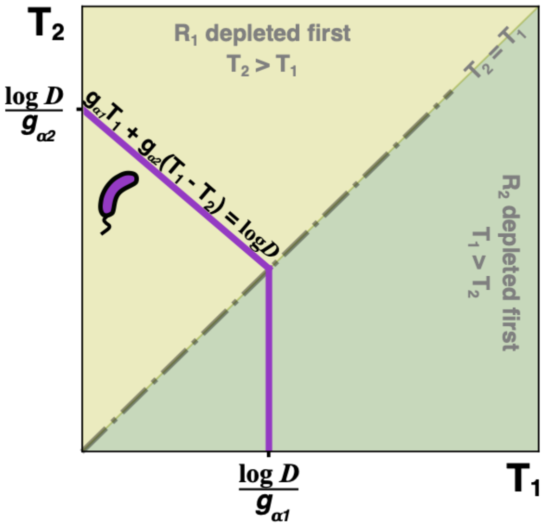

Supplementary Figure 9: **Tilman-inspired diagram for one microbe.** Zero Net Growth Isocline (ZNGI) of a single microbe B_α growing in an environment that contains two sources R_1 and R_2 with the growth rates $g_{\alpha 1}$ and $g_{\alpha 2}$ correspondingly. The environment is subject to serial dilution with the dilution coefficient D. The axes T_1, T_2 represent the corresponding depletion times of R_1, R_2. In the given environment, the microbe's top choice is R_1. If the latter is depleted last (the lower triangle of the diagram, $T_1 > T_2$), the microbe grows on it with the growth rate $g_{\alpha 1}$, never exhibiting a diauxic shift.

In the similar way, for the rest of the effective growth time $T_2 - T_1$, R_2 is consumed, and we get:

$$T_2 - T_1 = \frac{1}{g_{\alpha 2}} \log \Big(\frac{D(R_1 + R_2)}{DR_1 + R_2}\Big). \tag{S.7}$$

In the case when a single microbe resides in the environment containing two resources, the depletion order is defined by the microbe's preferences, and the steady state concentration of the microbe as well as the depletion times depend on the initial resource concentrations of each dilution cycle.

When a single microbe invades a multi-nutrient environment, the resources concentration values being $R_1, R_2, ..., R_k, ..., R_n$, the sequence in the above reflecting the preferential order of that particular microbe B_α, the depletion times are obtained in the way similar to the above, so that

$$\Delta T_k = T_k - T_{k-1} = \frac{1}{g_{\alpha k}} \log \Big(\frac{D(R_1 + R_2 + ... + R_{k-1} + R_k) + R_{k+1} + ... + R_n}{D(R_1 + R_2 + ... + R_{k-1}) + R_k + R_{k+1} + ... + R_n}\Big) \tag{S.8}$$

In the case of a balanced nutrient supply $R_1 = R_2 = ... = R_n$ and $D \gg n$ one can write

$$\Delta T_1 = T_1 \simeq \frac{1}{g_{\alpha 1}} \log \Big(\frac{D}{n}\Big),\ \Delta T_2 = T_2 - T_1 \simeq \frac{1}{g_{\alpha 2}} \log \frac{2}{1},\ \Delta T_3 = T_3 - T_2 \simeq \frac{1}{g_{\alpha 3}} \log \frac{3}{2} ... \tag{S.9}$$

so that $\Delta T_k = (1/g_{\alpha k}) \log(n/(n-1))$. So, at large values of the dilution coefficient D the microbe spends a huge share of the growing time consuming its top-choice resource. Its concentration gets multiplied by approximately D/n upon using up R_1, while consummation of R_2 just doubles it, and so on.

For a medium containing two resources R_1 and R_2, invaded by a single microbe B_α, we could construct a Tilman-like diagram (see Fig. 9). We have found that, for the serial dilution setting, the state-defining role of the resource concentrations assigned to the axes on the original Tilman's plane[2] should be played instead by the depletion times T_1 and T_2. Any point (T_1, T_2)

of the line described by the equation (S.2) represents the pair of depletion times in a steady state, determined by a particular set of the initial resource concentrations. The line is only valid when $T_2 > T_1$ — that would be the depletion order determined by the nutritional preferences of the microbe B_α.

When yet another microbe B_β tries to invade in a seed amount, the challenge is to survive and grow in the medium shaped for it by the microbe B_α. During the first dilution cycle at which B_β is present, as it has been pointed out in the main text, its influence on the resources lifetimes is negligible. Thus

$$g_{\beta 1}\, T_1 + g_{\beta 2}\, (T_2 - T_1) > \log D \tag{S.10}$$

($g_{\beta 1}, g_{\beta 2}$ being the new invader's growth rates on the resources R_1 and R_2) is the criterion of whether B_β is able to grow in the invaded medium **if the resources' ranks in its preferential list correspond to the depletion order of the resources in the invaded environment.**

For a microbe B_γ that prefers R_2 to R_1, the criterion of successful invasion of the environment shaped by B_α would be

$$g_{\gamma 2}\, T_2 > \log D \tag{S.11}$$

meaning that what matters is whether it can grow on its top-choice resource (of those present in the environment) fast enough.

The Tilman-inspired diagrams with the sole-microbe steady state ZNGI lines of B_β and B_γ, together with that of B_α, are featured in Fig.10. Each of them corresponds to the set of the steady states realizable under different nutrition conditions (i. e., the ratio of the concentrations R_1/R_2) when one microbe (B_α, B_β or B_γ) invades the abiotic environment.

In the usual way, the approach invented by Tilman enables one to determine whether a new-coming invader will survive under given circumstances. If the given steady state depletion times T_1 and T_2 define a point situated under the steady state lifeline (the ZNGI, to use Tilman's original terminology) of the invader, then the latter would not be able to grow. If, on the other hand, the depletion times characterizing the steady state map themselves to the point above the ZNGI of the invader, the latter will start growing and reshaping the environment until the depletion times set somewhere on its own ZNGI.

The numbers I, II, III in Fig.10 mark points in the Time-plane at which the coexistence of two microbes is possible. In absence of strong trade-off conditions there are, in general, no points at which three or more lines intersect, so that the number of survivors in a steady state can never exceed the number of nutrition sources. In presence of all the three contestants, the only possible coexistence point is II, at which the microbe B_α preferring R_1 to R_2 coexists with B_γ with the reversed preference order: the top choice R_2, the second choice R_1. In presence of the species B_γ the species B_α and B_β cannot coexist (at point I) as the diagram shows that, at the point I, B_γ can grow. The coexistence point III of the pair B_α, B_γ, on the other hand, is disabled by the presence of the species B_β whose ZNGI goes lower.

For the two species B_α and B_β to be able to coexist, we must have

$$\begin{cases} g_{\alpha 1}\, T_1 + g_{\alpha 2}\, (T_2 - T_1) = \log D \\ g_{\beta 1}\, T_1 + g_{\beta 2}\, (T_2 - T_1) = \log D \end{cases} \tag{S.12}$$

From that we get the depletion times that permit coexistence of B_α and B_β in absence of B_γ (point I in Fig.10)

$$\begin{cases} T_1 = \dfrac{g_{\beta 2} - g_{\alpha 2}}{g_{\alpha 1}\, g_{\beta 2} - g_{\beta 1}\, g_{\alpha 2}} \log D \\ \\ T_2 = \dfrac{g_{\alpha 1} - g_{\alpha 2} - g_{\beta 1} + g_{\beta 2}}{g_{\alpha 1}\, g_{\beta 2} - g_{\alpha 2}\, g_{\beta 1}} \log D. \end{cases} \tag{S.13}$$

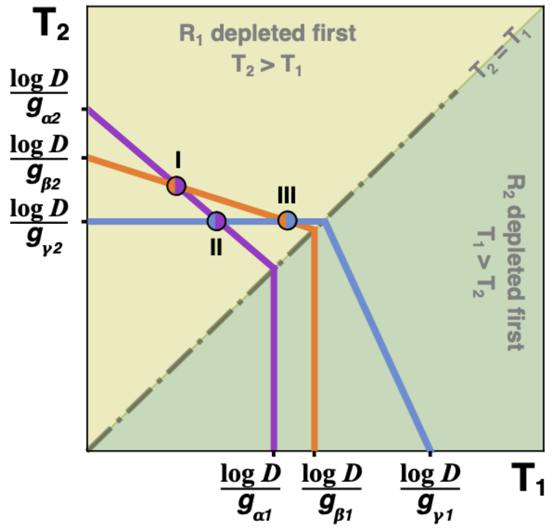

Supplementary Figure 10: **Tilman-inspired diagrams featuring possible coexistence of microbes with similar and different nutrition preferences.** The ZNGI lines of the microbes B_α (lilac) and B_β (orange) with the same top choice R_1 intersect at the point **I**, at which both can coexist within a certain range of the R_1/R_2 ratio. At that point however B_γ (light blue) with the top choice R_2 can invade, supplanting B_β and bringing the system to the coexisting point **II**. In absence of B_α the microbes B_β and B_γ are able to coexist at the point **III**.

To get the coexistence conditions for B_α and B_γ sharing the same environment (point II), as the coexistence happens at $T_2 > T_1$ and thus B_γ uses only its top choice R_2 through the whole growing period, we write

$$\begin{cases} g_{\alpha 1} T_1 + g_{\alpha 2} (T_2 - T_1) = \log D \\ g_{\gamma 2} T_2 = \log D \end{cases} \tag{S.14}$$

That leads to

$$\begin{cases} T_1 = \dfrac{g_{\alpha 2} - g_{\gamma 2}}{g_{\gamma 2} (g_{\alpha 1} - g_{\alpha 2})} \log D \\ \\ T_2 = \dfrac{\log D}{g_{\gamma 2}} \end{cases} \tag{S.15}$$

The coexistence point III at which B_β and B_γ can share the same environment in absence of the microbe B_α is defined by the formulae similar to (S.15), when obvious substitutions are made.

The conditions (S.13) and (S.15) show that the depletion times enabling coexistence (in the case of maximum diversity, when the number of microbes equals that of the sources of nutrition) are fully determined by the relevant growth rates. That kind of coexistence required the ratio of the resource concentrations R_1/R_2 to fall within a certain range determined by the growth rates and the dilution coefficient.

The steady state condition imposed on the initial (at the beginning of each dilution step) microbe concentrations in the case of B_α and B_β peacefully coexisting:

$$DN_\alpha + DN_\beta = R_1 + R_2 + N_\alpha + N_\beta \tag{S.16}$$

It T_1 is the depletion time of R_1 from (S.13), in the steady state in which B_α coexists with B_β one must have

$$N_\alpha \exp(g_{\alpha 1} T_1) + N_\beta \exp(g_{\beta 1} T_1) = R_1 + N_\alpha + N_\beta \tag{S.17}$$

Combining (S.16) and (S.13) to find steady state concentration values of B_α and B_β and requiring those to be positive, we get the condition

$$\frac{\exp\left(g_{\beta 1}\, T_1\right) - 1}{D - \exp\left(g_{\beta 1}\, T_1\right)} < \frac{R_1}{R_2} < \frac{\exp\left(g_{\alpha 1}\, T_1\right) - 1}{D - \exp\left(g_{\alpha 1}\, T_1\right)} \tag{S.18}$$

In Eq. (S.13) and (S.18) we implied that $g_{\alpha 1} > g_{\beta 1}$ and $g_{\alpha 2} < g_{\beta 2}$, as the diagram in the Fig.10 would have it. This is the usual counter-ordered arrangement for the coexistence of the species with similar preferences.

In the case of B_α and B_γ coexisting, their top choices being different, Eq. (S.17) relating the microbe and resource concentration values at the moment when R_1 (which is the first to disappear) is consumed, takes the form

$$N_\alpha \exp\left(g_{\alpha 1} T_1\right) = R_1 + N_\alpha \tag{S.19}$$

as the microbe B_β never tastes R_1. Obtaining the condition for B_β to be present in the steady state and thus have positive concentration value, we get simply:

$$\frac{R_1}{R_2} < \frac{\exp\left(g_{\alpha 1} T_1\right) - 1}{D - \exp\left(g_{\alpha 1} T_1\right)} \tag{S.20}$$

Comparing Eq. (S.18) with Eq. (S.20) we conclude that, generally speaking, the range of the ratio R_1/R_2 values favoring coexistence is larger when the microbes in question have complementary preferences.

Note that in the above case **the concentrations** (R_1, R_2) **do not map point to point onto the Time-plane**. In the case of the coexistence of two microbes, be it the species with the similar or different nutrition preferences, a single point (T_1, T_2) corresponds to a continuous interval of the ratio R_1/R_2, or, in the other words, to a band with non-parallel straight boundaries in the original Tilman's resource plane.

The depletion order in the case of two resources present in the environment, barring special cases, is defined by the absolute fastest consumer of its top-choice resource. Indeed, if the top-choice of such a fastest-growing microbe were the last to disappear, the champion microbe would grow on it through the whole dilution cycle and thus its concentration would acquire greater multiplication coefficient than that of any other competing microbe. So it will be the absolute winner — yet, in this case, it will shape the environment in such a way that its top-choice resource is the first to disappear. For the same reason, when the resources present in the environment are multiple, the absolute fastest grower will not have its top-choice resource to be the last to disappear, again barring certain special circumstances. In the multi-resource environment, however, the champion species cannot be expected to establish the depletion order all on its own.

Now to the special circumstances from the previous passage. Those have to do with the special species, in the main text referred to as **anomalous** (as opposed to **normal**) microbes. In the case of an environment containing just two resources, the anomalous microbes are those that grow faster on their second choice resource, and not on their top choice one. Such microbes can display a bizarre nutrition behaviour, as can be seen in the Fig. 11.

An anomalous microbe is easily distinguished on a Tilman-inspired diagram by the different slope, rising up from the relevant time-axis instead of falling to it, as is the case with the normal microbes' ZNGI. It turns out that even being the absolute fastest grower (but on its second choice!), such a species may not be able to invade an environment if its top choice (on which it grows slower) is the last to disappear. In presence of an anomalous species, bistability may arise. In the case shown in Fig. 11, the resulting steady state will depend on which species, the anomalous one or its normal counterpart, is the first to invade. The anomalous species can make life in a biocontainer less dull by setting off the predetermination; more of that in the section dedicated specially to the anomalous microbes and the reason of them being rare.

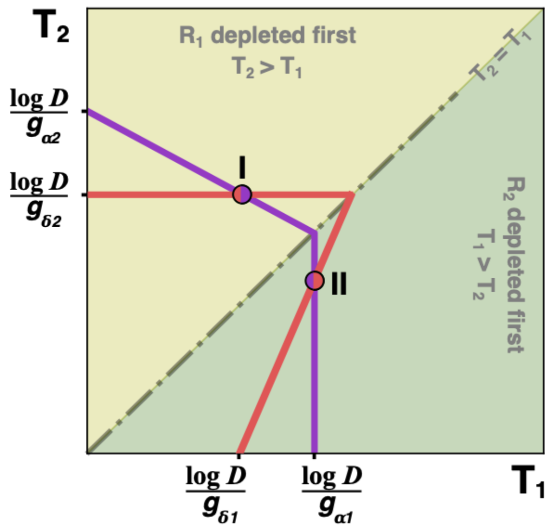

Supplementary Figure 11: **An anomalous microbe competing with a normal one, represented by a Tilman-inspired diagram.** The anomalous microbe B_δ with the top choice R_2 (red ZNGI, with the positive slope at $T_1 > T_2$ displaying the abnormality) can coexist with the normal microbe B_α (lilac ZNGI, top choice R_1) at the points **I** and **II**. The coexistence at **I** is stable, while the coexistence at the point **II** is unstable. If prepared, the latter decays either down to the state at which B_δ (or B_α) is the single resident, or up to the point of the stable coexistence **I**, depending on the value of the ratio R_1/R_2. Within a certain region of the values of R_1/R_2 bistability can occur. If B_α is the first to invade, it may either withstand the invasion by B_δ or let it share and coexist, depending on the value of R_1/R_2. If B_δ is the first to invade, it may shape the environment so that B_α cannon invade in its turn. Thus, depending on the order of invasion, the bistability between two different final steady states may be possible. Further investigation shows that, depending on the dilution coefficient and the growth rate parameters, the bistability may occur either between two monoculture states or between two states one of which is that of monoculture and the other is that of coexistence.

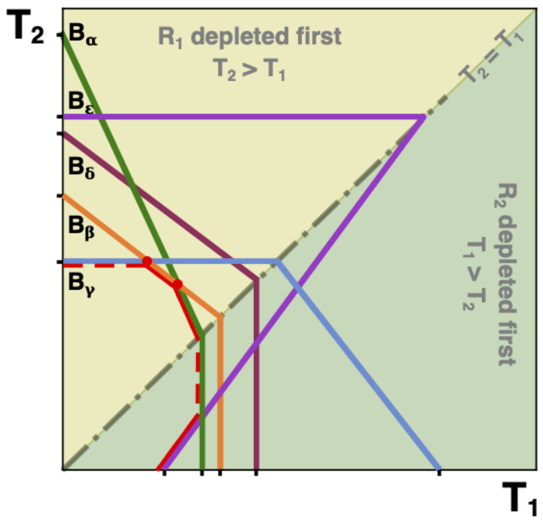

Supplementary Figure 12: **The Pareto-optimal boundary.** Of all the competing microbes, each one except B_δ (auburn ZNGI, top choice R_1) participates in the forming of the Pareto-optimal boundary (marked in red). The parts of the POB marked by the dashed red line do not contain any points that correspond to the realizable steady states of the system. They are useful however to determine whether, for instance, B_γ (light blue ZNGI, top choice R_2) can invade an environment shaped by, say, B_β (orange ZNGI, top choice R_1) at a given steady state. When it can, the coexistence with B_β will occur. As the point of coexistence belongs to the Pareto-optimal boundary, such a state will be uninvadable. At the balanced nutrient supply, the steady state of the system will most likely correspond to a point on the POB not far from the diagonal $T_1 = T_2$.

B Pareto-optimal boundary and the one-microbe line

B.1 Relation to Tilman's graphical method

The original Tilman's diagrams that have resource concentrations for the axes enable one to predict which steady states can be uninvadable by visual means[3, 4]. For that, two things must appear on the diagram: first, the full set of the life vs death boundaries (ZNGI) for each species, to determine which species can grow when the others would die out; second, the so-called one-microbe lines. Each of the latter is the dynamic trajectory of a single-species state, traced from a particular abiotic point to that same species' zero net growth rate isoclines; at the intersection, a steady state arises. Usually the ZNGI lines are fully determined by the properties of the species they correspond to (e. g. growth rates, yields, maintenance costs etc.) and the dilution coefficient when relevant, while the one-microbe lines depend on the properties of a particular species as much as on the external conditions.

Our Tilman-inspired diagrams are a no less powerful instrument in this sense, to be handled with care, as, in general, they do not map to a resource plane (or space) point by point. In the serial dilution case, the ZNGI lines get scaled by the dilution coefficient D; otherwise, they are completely determined by the growth rates of the corresponding species.

B.2 The Pareto-optimal boundary

When there are many competitors in the environment, some of the species never get to survive, and some others can win the game only at exotic external conditions (e. g. large imbalance in the regular nutrient supply). The question of which of the species can boast a range of external conditions favorable to their survival at the expense of the other competitors is related to the notion of *Pareto-optimal boundary.*

A ZNGI line for a microbe B_α that prefers the resource R_1 to R_2 has the equation

$$g_{\alpha 1} T_1 + g_{\alpha 2}(T_2 - T_1) = \log\, D, \tag{S.21}$$

in the upper segment of the positive quarter of the Time plane T_1, T_2: here $T_2 > T_1$ so that B_α starts with R_1, finishes it till the end of the time period T_1 and then, for the time period of the length $T_2 - T_1$ grows on the resource R_2. Whenever it grows more than $D-$fold through a dilution cycle (i. e. the time point of the environment is positioned above that boundary), it survives; if however the times of the environment correspond to a point below the line (S.21), the species B_α is doomed to get scarce and vanish from the serially diluted environment. In the lower segment of the positive quarter of the Time plane we have $T_1 > T_2$, so that through the whole effective growth time of the dilution cycle R_1 is present and B_α does not have to switch eventually to R_2. The segment of the life vs death boundary for B_α belonging to this area has the equation

$$g_{\alpha 1} T_1 = \log\, D. \tag{S.22}$$

The latter segment does not account for any single-microbe steady state point due to the fact that, when single, B_α will eventually shape the environment in such a way that R_1 is depleted first. Still, it is useful to judge whether B_α can grow when it invades the environment in which R_1 is the last to be depleted, and whether it can possibly coexist with another microbe in such an environment.

In Fig. 12 five microbes B_α, B_β, B_γ, B_δ, B_ϵ compete for the resources R_1, R_2. The shapes of their ZNGI give one a hint that B_α, B_β, B_δ prefer R_1 to R_2 while B_γ and B_ϵ choose otherwise. It is also clear that B_ϵ is anomalous, meaning that when given a choice, it starts consuming R_2 rather than R_1 despite the fact that it grows faster on R_1. (It is useful to remember that the ZNGI lines hit the axes T_1 and T_2 at the points $(\log D)/g_1$, $(\log D)/g_2$ respectfully, so that the closer the point is to origin, the larger the corresponding growth rate.) **The lower envelope** of the zero net growth isoclines is the line indicating the winners of the game (either single species or coexisting pairs) under various external conditions. We refer to it as the **Pareto-optimal boundary**. In Fig. 12 it is drawn in red. The dashed segments of the Pareto-optimal boundary trace the depletion times that effectively cannot be realized in a steady state; any steady state which is uninvadable can be represented by a point on the solid red line.

B.3 One-microbe line

Now to the notion of the one-species or the one-microbe line. While in the models considered by Tilman[3, 4] the resources are being continuously depleted, and for that reason the trajectories in the resource plane are continuous, with the depletion times in the serial dilution experiment that cannot be the case. If, say, B_α is introduced to an abiotic environment of a serial dilution experiment with the dilution coefficient D and initial nutrient abundances R_1, R_2 in a seed amount, it may not be able to consume the available amount of R_1 through the first dilution cycle, or even a couple of those. While this is the case, the depletion times remain $T_1 = T_2 = T$, where T is the fixed durance of the dilution cycle, and the "one-microbe line" is reduced to a single point. When B_α is finally able to consume R_1, it may or may not manage to deplete R_2 during the cycle; if the latter is the case, T_1 diminishes, while T_2 remains equal to the durance of the cycle, so the point representing the state of the system in terms of the depletion times travels left along the line $T_2 = T$. When B_α, finally, succeeds at depleting both R_1 and R_2 in one dilution cycle, we would have, for some dilution cycle number k:

$$B_\alpha^{(k)}\, \exp\left(g_{\alpha 1} T_1^{(k)}\right) = R_1 + B_\alpha^{(k)} \tag{S.23}$$

and

$$B_\alpha^{(k)}\, \exp\left(g_{\alpha 1} T_1^{(k)} + g_{\alpha 2}\left(T_2^{(k)} - T_1^{(k)}\right)\right) = R_1 + R_2 + B_\alpha^{(k)}, \tag{S.24}$$

where the upper index (k) means that value relevant for the $k-$th dilution cycle.

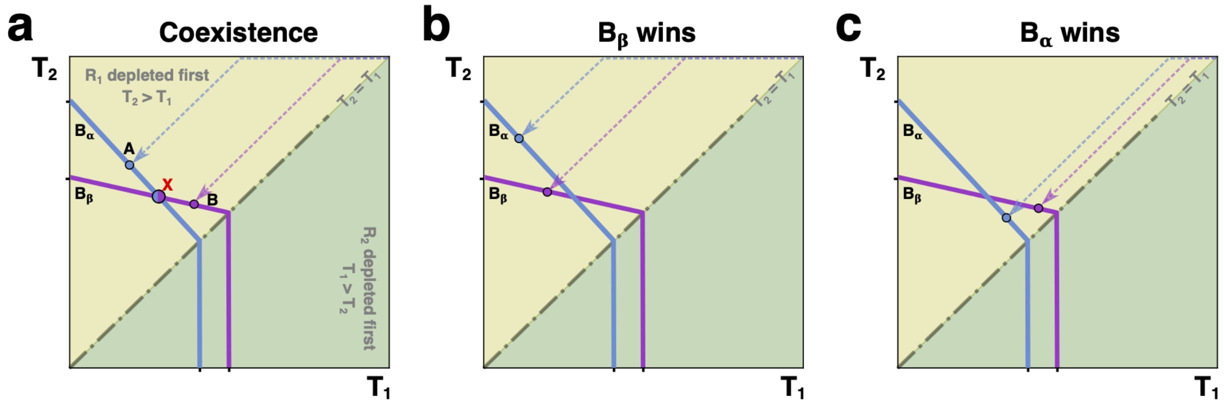

Supplementary Figure 13: **The one-microbe lines indicating the winner(s). (a)** The (light blue, dotted) one-microbe line of the microbe B_α lands on the (light blue, solid) ZNGI of that same microbe at the point **A**. At this point, the species B_β can invade. The (lilac, dotted) one-microbe line of the bacterium B_β lands on the (lilac, solid) ZNGI of that species at the point **B**. At this point, the competing microbe B_α can invade. As a result of both invasions, the species B_α and B_β will coexist at the point **X**. **(b)** The (light blue, dotted) one-microbe line of B_α lands on the (light blue, solid) ZNGI of that same species at the point where B_β can invade. The (lilac, dotted) one-microbe line of B_β lands on the species' ZNGI (lilac, solid) at the point where B_α cannot invade. Thus B_β wins the competition. **(c)** The (light blue, dotted) one-microbe line of B_α lands on the species' ZNGI (light blue, solid) at the point where B_β cannot invade. The (lilac, dotted) one-microbe line of B_β lands on the (lilac, solid) ZNGI of that same species at the point where B_α can successfully invade. In this case, B_α is the sole winner.

Excluding $B_\alpha^{(k)}$ from both (S.23) and (S.24), we get

$$T_2^{(k)} = T_1^{(k)} + \frac{1}{g_{\alpha 2}} \log\left(\frac{R_2}{R_1}\left(1 - \exp\left(-g_{\alpha 1} T_1^{(k)}\right)\right) + 1\right) \quad \text{(S.25)}$$

At $g_{\alpha 1} T_1^{(k)} \gg 1$ which is certainly true for reasonably balanced nutrient supply and $D \gg 1$, through the whole dynamics from that point on we would get $T_2 \sim T_1 + \text{const}$, so that the one-microbe line would go almost parallel to the diagonal $T_1 = T_2$. It is not continuous, so in reality the one-microbe line of B_α is a set of points belonging to the curve from (S.25). At the steady state $g_{\alpha 1} T_1 + g_{\alpha 2}(T_2 - T_1) = \log D$, so that using (S.25) we get for the steady state depletion times:

$$T_1 = \frac{1}{g_{\alpha 1}} \log\left(\frac{DR_1 + R_2}{R_1 + R_2}\right), \; T_2 = T_1 + \frac{1}{g_{\alpha 2}} \log\left(\frac{D\,(R_1 + R_2)}{DR_1 + R_2}\right), \quad \text{(S.26)}$$

same as we obtained in the previous sections.

In the same way as in bona fide Tilman's diagram, the species B_α can coexist with the species B_β if the one-microbe line of B_α ends at the point at which B_β can grow, and vice versa (see Fig. 13, panel A). If however the microbe's one-microbe line lands on the ZNGI at a point where for any other competitor the growth is impossible, it becomes the sole winner (barring the bistability cases), as it is shown on the panels B, C Fig. 13. Note that the one-microbe line of B_β always lands on the microbe's ZNGI at larger values of T_1, as its growth rate $g_{\beta 1}$ is smaller, and thus its $T_1 = (1/g_{\beta 1}) \log\Big((DR_1 + R_2)/(R_1 + R_2)\Big)$ is larger than the depletion time of R_1 from (S.26). This is why the one-microbe line of B_β is always shown on the right of that of B_α. (Remember that we consider yields of different microbes wrt different sources to be equal. If they are not equal, the one-microbe lines will display more complex behavior, see the corresponding SI section.)

Another thing worth mentioning is that T_1 in Eq. (S.26) obviously grows larger with the increase of the ratio R_1/R_2, thus moving the steady state point down the slope (or up the slope,

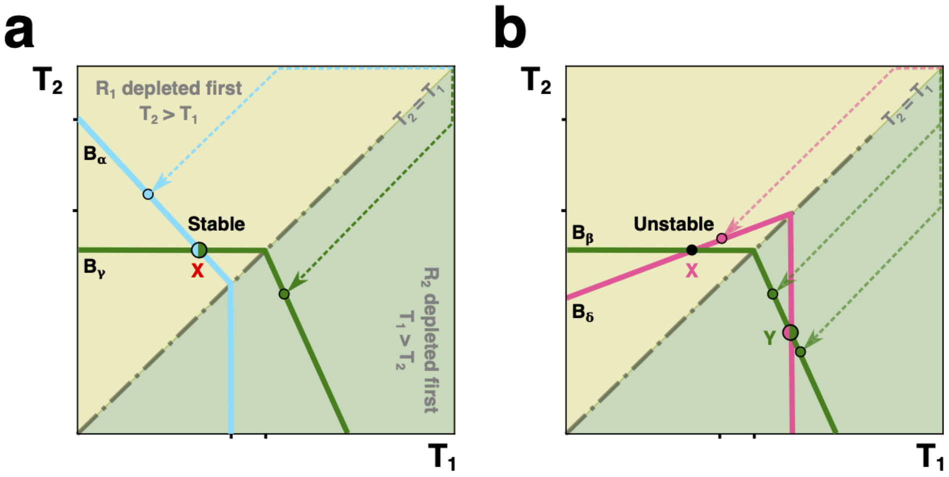

Supplementary Figure 14: **Stable (a) and unstable (b) coexistence.** **(a)** The (light blue, dotted) one-microbe line of the bacterium B_α lands on its (light blue, solid) ZNGI at the point where B_γ can grow. The (green, dotted) one-microbe line of the microbe B_γ lands on its (green, solid) ZNGI at the point where B_α is able to grow. The invasion of the environment by both microbes will result in stable coexistence. **(b)** The (pink, solid) ZNGI of the anomalous microbe B_δ (top choice R_1) intersects with the (green, solid) ZNGI of the normal microbe B_β (top choice R_2) at points **X** above the diagonal $T_1 = T_2$ and **Y** below the diagonal. To achieve coexistence, the one-microbe lines of each microbe should land where the other one can grow. The one-microbe line of the anomalous B_δ (pink, dotted) lands on the microbe's ZNGI (pink, solid) at a point where B_β can grow. If the (green, dotted) one-microbe line of B_β lands on its (green, solid) ZNGI above the point **Y**, where the anomalous B_δ cannot grow, then the normal B_β comes out a sole winner. If the one-microbe line of B_β lands on its ZNGI below the point **Y**, where B_δ can grow, both species would end up coexisting at the point **Y**. The coexistence at the point **X** thus cannot be realizable by competition, and is unstable if prepared.

if the microbe is anomalous) of the corresponding ZNGI in the Time plane.

B.4 Types of the steady state coexistence: stable and unstable

The coexistence shown in Fig. 13 is obviously stable as no single one of the given two microbes competing for the resources cannot on its own supplant another. The same goes for the coexistence of the species B_α and B_γ that have different preferences with regard to the resources R_1 and R_2, see Fig. 14, panel A. Coexistence at the point X is stable when the one microbe line of B_α hits it ZNGI above the point X (and thus the zero net growth isocline of its rival B_γ goes under that of B_α) and the same is true for the one microbe line of B_γ, so that at the point of its own single-microbe steady state it cannot supplant B_α. This (panel A) is the case when both microbes are normal. (One is reminded that we consider a microbe "normal" if it grows fastest on its top choice. Otherwise, the species is regarded as"anomalous".) On the contrary, when the anomalous species enter the picture (Fig. 14, panel B), some steady states that one can technically prepare turn out to be unstable. For instance, the coexistence of the microbes B_γ (normal) and B_δ (anomalous) at the point X (Fig. 14, panel B) would not work; if prepared, it will degenerate either into the coexistence point Y, if the one-microbe line of B_γ goes the way the solid version (1) does, landing on its own ZNGI where B_δ can grow, or else into the single-microbe steady state of B_γ if the latter's one-microbe line goes like the dashed version (2). (If the one-microbe line of B_δ lands to the left of the point X, then B_δ is the sole winner.) That stands to reason from the physical point of view. Prepare the steady state at the point X and disturb the environment, say, by adding a small amount of the species B_γ making its abundance a little larger than that required for the steady state. That would not affect at first the depletion time T_1 of R_1 as B_γ does not consume it in the state in question, yet the depletion time T_2 in the dilution cycle during which the abundances got disturbed will

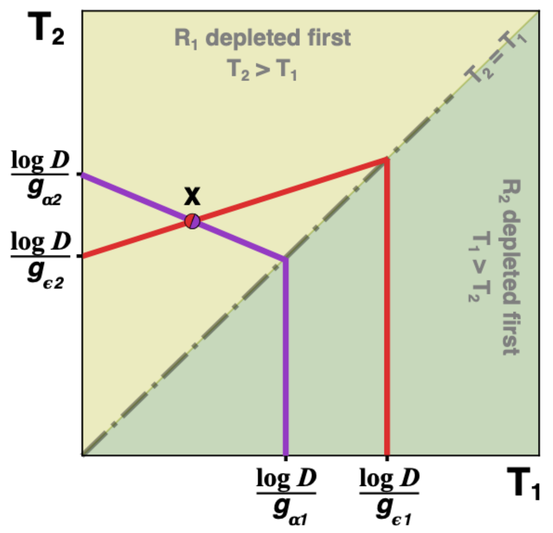

Supplementary Figure 15: **An anomalous species coexisting with a normal one with the same nutrition preferences.** Within a particular range of the values of the ratio R_1/R_2 the anomalous bacterial species B_ϵ (the red ZNGI with the "anomalously" positive slope above the diagonal $T_1 = T_2$) can coexist with the normal microbe B_α (the lilac ZNGI with the "normal" negative slope above the diagonal) at the point **X**.

be shorter, as there is more of B_γ to use it up. As a consequence, B_δ will come out of it grown less than $D-$fold, and for the next cycle its abundance will be less than the steady state amount. So it will be slower yet to consume R_1 at which it grows less fast than at R_2 being anomalous, and B_γ will have more time to grow on its favored resource R_2 all on its own. Thus B_δ gets lesser share of R_2 that is essential for its growth and loses the next dilution cycle, and so on. Eventually the depletion order changes to first R_2, then R_1, and the outcome depends on whether B_γ can supplant B_δ (dashed line) or will have to share (solid line in Fig. 14, panel B). This is determined by the ratio R_1/R_2 (see e. g. eq. S.25 or the discussion in previous sections).

C Anomalous microbes and their rarity

In this section we discuss the anomalous species. Generally speaking, if a bacterium B_ϵ with a diauxie growth curve prefers the resource R_i to the resource R_j despite the fact that it grows faster on the less-preferred nutrient, $g_{\epsilon i} < g_{\epsilon j}$, we consider it anomalous.

The definition would stay rather vague unless we claim the absolute knowledge of the preference list of a species in its totality. Effectively, we would deal with a limited number of resources and will probably not notice any effect of a species being anomalous unless it is its top choice (among the nutrients available) that provides the microbe with a lesser value of the growth rate than the second-best. This is the context in which the term "anomalous" will be used further on, the more so as, in this section, we deal mostly with an artificial environment containing just two resources.

In our simulations, using the balanced nutrient supply (meaning that the concentrations of the resources are equal at the beginning of each dilution cycle) and the values of the dilution coefficient D varying from 2 to 10000, we find the anomalous microbes vanishing from the steady state community of survivors as long as the latter becomes more and more mature.

To see why that happens, consider point X in Fig. 15, which is a point in the Time plane at which the microbe B_α that is normal and prefers R_1 to R_2 coexists with the microbe B_ϵ which is anomalous, although it shares the former's preferences. If in the steady state $T_1 < T_1(X)$,

the anomalous species is the winner, and we are interested in the values of the ratio R_1/R_2 and the dilution coefficient D at which that happens. Note that $g_{\epsilon 1} < g_{\alpha_1}$ meaning that the anomalous microbe we consider here does not excel at both growth parameters, only at that of its second choice. Thus our case has nothing to do with the "superbug" event, we consider a regular situation.

Consider $\Delta T_1 = T_1 - 0$ and $\Delta T_2 = T_2 - T_1$, the actual lifetimes of the resources R_1 and R_2 from the moment they, each in its turn, are attacked by the consuming microbe(s). If B_ϵ is a single tenant that had established the steady state and B_α is invading, the latter cannot survive when

$$g_{\epsilon 1}\,\Delta T_1 + g_{\epsilon 2}\,\Delta T_2 > g_{\alpha 1}\,\Delta T_1 + g_{\alpha 2}\,\Delta T_2. \tag{S.27}$$

$\exp(g_{\epsilon 1}\Delta T_1) = R_1 + N_\epsilon$ for the moment when R_1 is totally consumed, N_ϵ being the steady state concentration of the bacterium B_ϵ at the beginning of each dilution cycle, and the steady state multiplication rule $g_{\epsilon 1}\Delta T_1 + g_{\epsilon 2}\Delta T_2 = D$, provide us with the values of the effective times of consummation for both resources:

$$\begin{cases} \Delta T_1 = \dfrac{1}{g_{\epsilon 1}}\,\log\Big(\dfrac{DR_1 + R_2}{R_1 + R_2}\Big) \\[2ex] \Delta T_2 = \dfrac{1}{g_{\epsilon 2}}\,\log\Big(\dfrac{D\,(R_1 + R_2)}{DR_1 + R_2}\Big) \end{cases} \tag{S.28}$$

Combining (S.27) and (S.28) we get

$$\frac{g_{\alpha 1} - g_{\epsilon 1}}{g_{\epsilon 1}}\,\log\Big(\frac{DR_1 + R_2}{R_1 + R_2}\Big) < \frac{g_{\epsilon 2} - g_{\alpha 2}}{g_{\epsilon 2}}\,\log\Big(\frac{D\,(R_1 + R_2)}{DR_1 + R_2}\Big). \tag{S.29}$$

As long as (S.29) holds, the normal microbe B_α cannot invade the environment shaped by the anomalous microbe B_ϵ. To get the criterion for the event of B_ϵ withstanding the invasion in a convenient form, we transform (S.29) and introduce a parameter

$$y = \Big(\frac{g_{\epsilon 2}(g_{\alpha 1} - g_{\epsilon 1})}{g_{\epsilon 1}(g_{\epsilon 2} - g_{\alpha 2})} + 1\Big)^{-1} = \frac{g_{\epsilon 1}\,(g_{\epsilon_2} - g_{\alpha_2})}{g_{\epsilon 2}g_{\alpha 1} - g_{\epsilon_1}g_{\alpha 2}} < 1. \tag{S.30}$$

We get the criterion

$$\frac{R_1}{R_2} < \frac{D^y - 1}{D - D^y} \tag{S.31}$$

The criterion (S.31) can mean either a huge imbalance in terms of the nutrient supply or something reasonable, depending on the values of y and D.

Using elementary calculus, we conclude that the right hand part of the equation is a decreasing function of D, at least when it goes all the way from 2 to ∞, at any fixed y.

While at $D = 2$ it is possible to get the left-hand part of (S.31) well over $R_1/R_2 = 1$ at reasonable values of y, there is yet another factor coming into the game, closely related to the rise of complementarity at any value of D. Consider the diagram from Fig. 15 with the addition of yet another species B_γ that prefers R_2 to R_1 and is a capable consumer of its top choice.

In Fig. 16 the green species B_γ invades the environment shaped by the coexisting B_α the normal and B_ϵ the anomalous microbes. The invader is perfectly able to grow as its lifeline passes below X that is the point of the said coexistence. When B_ϵ grows, it affects T_2 that is becoming less, meaning that the microbes B_α and B_ϵ start each dilution cycle in smaller amounts and T_1 becomes larger. That shifts the position of the system towards the point Z of the coexistence of two normals B_α and B_γ that have different nutrition preferences.

One might wonder whether the complementarity manifests itself at all at the smaller values of the dilution coefficient D. The question is closely related to the crucial importance of the top choice nutrient and the value of the growth rate of the microbe associated with it. One of our arguments for the top choice being the most important from the very beginning was that

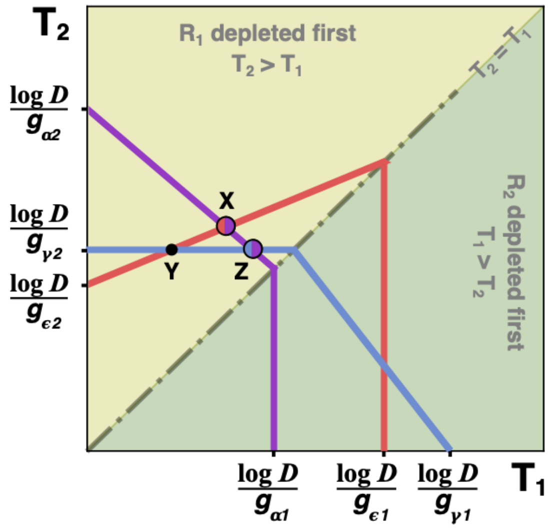

Supplementary Figure 16: **The coexistence of the anomalous species B_ϵ with the normal species B_α with the same preferences disabled by an invasion of the microbe B_γ with the reversed preferences and thus giving way to the complementarity.** An anomalous microbe B_ϵ (red ZNGI with the positive slope above the diagonal $T_1 = T_2$, top choice R_1) can coexist with a normal microbe B_α (lilac ZNGI, top choice R_1) at the point **X** in a stable manner. However, the microbe B_γ (light blue ZNGI, top choice R_2) with the complementary food preferences can invade at that point and supplant the anomalous microbe, switching the state of the system to the point **Z** at which the normal microbes B_α and B_γ share the environment. The coexistence point **Y** of B_γ and B_ϵ is unstable.

in order to reach the steady state, a species must have its initial concentration multiplied by D towards the end of each dilution cycle, and most of that multiplication is done at the expense of the first nutrient it consumes. Indeed, the steady state value of the microbe concentration when it is the single tenant of the environment being $\sum R_i/(D-1)$, which is also $nR/(D-1)$ when all the n resources are present in equal concentrations, is much less than R if $n \ll D$. So, upon consuming its top choice, a nutrient has its concentration multiplied by $(D-1)/n$ which is still a huge number, while the second nutrient in its turn multiplies it by less than 2, and each of the next-in-line resources does even poorer job at multiplying. On the contrary, at $D = 2$, the first resource augments the initial concentration of the microbe by a fraction $1/n$. While the second nutrient provides even less than that in terms of multiplication, that particular argument in favor of the top choice being the most important one becomes much less convincing.

What matters for the comparative importance of the first, second and further choices, as well as for the rise (or fall?) of complementarity, is the fact that the nutrient which is the last to be depleted remains the most reasonable target for invasion. Moreover, in the case of maximum diversity of the steady state community, complementarity is either already present or can be produced by an invasion with the probability that does not depend on D but rather is determined by the properties of the particular growth rate distributions. In the case of two nutrients, the equilibrium differential times are determined by the system of equations:

$$\begin{cases} g_{\alpha 1}\,\Delta T_1 + g_{\alpha 2}\,\Delta T_2 = \log D \\ g_{\beta 1}\,\Delta T_1 + g_{\beta 2}\,\Delta T_2 = \log D \end{cases} \tag{S.32}$$

(which in the general case would contain n equations for n unknowns), the solution would obviously not depend on the nutrient concentrations, and the condition for an invader B_γ

whose top choice is R_2 being able to successfully invade would have the form:

$$g_{\gamma 2} > \frac{g_{\alpha 1}\, g_{\beta 2} - g_{\alpha 2}\, g_{\beta 1}}{g_{\alpha 1} - g_{\alpha 2} + g_{\beta 2} - g_{\beta 1}} \tag{S.33}$$

It can be easily seen that the invader having its $g_{\gamma 2}$ equal (or less than but close) to $g_{\alpha 1}$ is the definite winner. Substituting the index β with ϵ we get the condition under which the invader B_γ chases away the anomalous species B_ϵ. The anomaly of B_ϵ, if anything, further frees the value of $g_{\gamma 2}$. (Of course, when $g_{\gamma 2} > g_{\alpha_1}$, the invader wins hands down, but in this case the depletion order will get reversed, and we would have to consider the anomalous species with the same preferences as B_γ all over again.)

To summarize, the anomalous species get driven to extinction at any values of the dilution coefficient D barring the cases of a huge imbalance in the nutrient supply, and the complementarity in a mature steady state also persists regardless of whether D is 2 or 10000.

D Multistability in a community of diauxic shifters

D.1 What prompts multistability

In some cases more than one uninvadable steady state is possible for the same initial conditions. Depending on the order of invasions, the system in the Time plane (or Time space, when the sources of nutrition are multiple) might end up at different points of the Pareto-optimal boundary. In the models associated with the continuously diluted chemostat, the phenomenon of multistability has been known to occur[5, 6]. With the co-utilisers, it comes to light when different yields of different microbes using the same resource are taken into account. When one deals with the diauxic shifters, however, the conflict of the preference lists of microbes and the rank lists of their growth rates, in other words, the presence of the anomalous microbes, is enough to produce multistability.

D.2 An example of bistability between two single-microbe states

In the case when only two microbes are present in the environment, bistability can occur. The reason why multistability can arise is easily seen by considering that simplest example.

In Fig. 17 (a) the black arrow points at the place at which the single-microbe state I of the (normal) microbe B_α is located on its zero net growth isocline (also black), and the green arrow marks the place at which the single-microbe state II of the (anomalous) microbe B_β hits its own (green) ZNGI. When B_α has shaped the environment in its single-microbe steady state I, B_β cannot invade, and vice versa.

To get the condition under which that kind of bistability can be realized, write for the environment shaped by B_α (with the depletion times $T_1^{(\alpha)} < T_2^{(\alpha)}$, $T_1^{(\alpha)} = (1/g_{\alpha 1}) \log\left((DR_1 + R_2)/(R_1 + R_2)\right)$, $T_2^{(\alpha)} = T_1^{(\alpha)} + (1/g_{\alpha 2}) \log\left(D(R_1 + R_2)/(DR_1 + R_2)\right))$

$$g_{\beta 2}\, T_2^{(\alpha)} < \log D, \tag{S.34}$$

and for the environment shaped by B_β

$$g_{\alpha 1}\, T_1^{(\beta)} < \log D, \tag{S.35}$$

with the depletion times $T_1^{(\beta)} > T_2^{(\beta)}$, $T_2^{(\beta)} = (1/g_{\beta 2}) \log\left((DR_2 + R_1)/(R_1 + R_2)\right)$, $T_1^{(\beta)} = T_2^{(\beta)} + (1/g_{\beta 1}) \log\left(D(R_1 + R_2)/(DR_2 + R_1)\right)$.

From (S.34, S.35) we get the bistability condition:

$$\frac{R_1}{R_2} > \max\left(\frac{D^x - 1}{D - D^x}, \frac{D - D^y}{D^y - 1}\right), \tag{S.36}$$

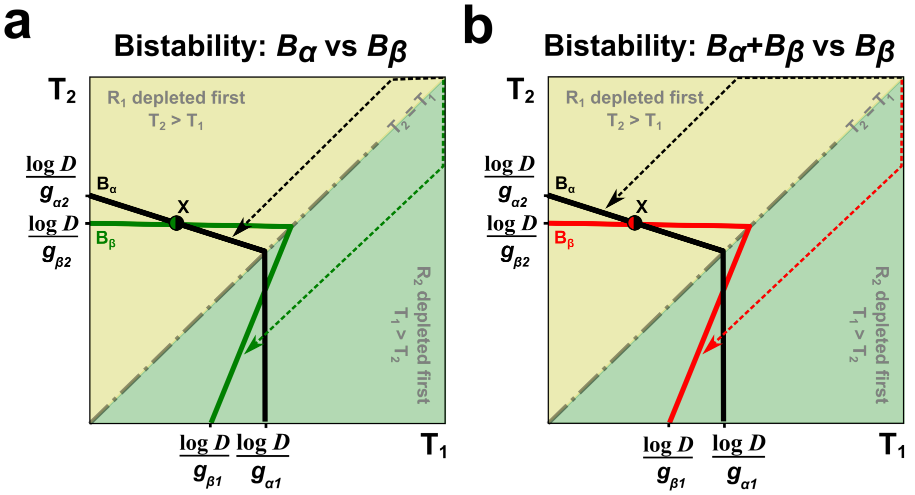

Supplementary Figure 17: **Bistabilities.** **(a)** The (black, dotted) one-microbe line of B_α lands on the microbe's (black, solid) ZNGI at the point where the bacterium B_β cannot grow. The (green, dotted) one-microbe line of B_β land on its (green, solid) ZNGI where the species B_α cannot grow. The resulting steady state will depend upon which microbe was the first to invade, hence there is bistability between two possible outcomes with different depletion orders. **(b)** The (dotted, red) one-microbe line of B_β lands on the microbe's (solid, red) ZNGI at the point where B_α cannot grow. The (dotted, black) one-microbe line of B_α lands on its (solid, black) ZNGI at the point where B_β can grow on its top choice, never displaying a diauxie shift. The resulting steady state will depend upon which microbe was the first to invade. If the bacterium B_α comes first, then, upon the invasion of B_β, the system acquires the steady state with both bacteria coexisting at the point **X**. If the microbe B_β is the first to invade, it will remain the sole resident. Hence there is bistability between two possible outcomes with different depletion orders and different degrees of diversity.

where

$$x = \frac{g_{\alpha 1}}{g_{\beta 2}} \frac{g_{\beta 2} - g_{\alpha 2}}{g_{\alpha 1} - g_{\alpha 2}}, \tag{S.37}$$

and

$$y = \frac{g_{\beta 2}}{g_{\alpha 1}} \frac{g_{\beta 1} - g_{\alpha 1}}{g_{\beta 1} - g_{\alpha 2}}. \tag{S.38}$$

D.3 An example of bistability between coexistence and exclusion for two microbial species

If the anomalous species B_β is very good at consuming both nutrients, the bistability may occur between the state at which both B_α and B_β are present peacefully coexisting — and the state at which it's B_β alone that has won the game.

Such a situation is presented in Fig. 17 (b). Here, the condition under which that bistability can be realized is more tricky

$$\frac{D^x - 1}{D - D^x} > \frac{R_1}{R_2} > \frac{D - D^y}{D^y - 1}, \tag{S.39}$$

with x, y the same as above. That implies $(D^x - 1)/(D - D^x) > (D - D^y)/(D^y - 1)$, i. e. $D^x + D^y > D + 1$. At least one of x, y would have to be rather close to 1, to make that happen.

D.4 Conclusions

In the line of thought of a recent work[7] that names conflicts as the source of complexity, in our system multiple steady states at the given external conditions occur when there is a conflict between the preference order inherent to a microbe and the rank order on its growth rate. We do not see much of that in our simulations of the environment with the balanced nutrient supply, although with an anomalous "super-bug" in the pool that could be arranged.

E On the attainability of the final steady state: does the invasion process end?

There is yet another complication to our model of the serial invasion related to the anomalous species. Why should the final steady state, uninvadable by any microbe from a given pool, even exist? We could have famous "rock-paper-scissor" cycles or stochasticity, even though we have never observed them in our simulations. In this section, we address two questions: (1) whether the situations in which the invasion process never ends can be arranged within the frames of our model; (2) if the answer is positive, why then do we always get a final and uninvadable state in our simulations.

E.1 An example of a cyclic final solutions

When points of unstable coexistence like X in Fig. 14 (b) appear on the Pareto-optimal boundary, they become "percolation points" of sorts. The dynamic trajectory of the system might "flow" from them over the line $T_1 = T_2$. Indeed, when B_γ invades the environment shaped by B_α (Fig. 14 (a)), it gets arrested at the point X, and the depletion order remains first R_1, then R_2, while invading the environment shaped by B_δ (Fig. 14 (b)) it either cannot survive or, never getting stuck at the point X, brings the system to some point where $T_1 > T_2$. That is almost of no consequence in the case of the environment containing just two resources, but when there are more of those, all the relevant points of coexistence, technically, can be unstable. When the latter is the case, we might see cycles or even stochasticity instead of the final uninvadable steady state.

An example of a cycle of the rock-paper-scissors type can be given for an environment containing three sources R_1, R_2, R_3, say, in equal abundances $R_1 = R_2 = R_3 = R$. Take three anomalous species B_ϕ, B_ψ, B_χ differing in their preferences only, so that $g_{\phi 1} = g_{\psi 2} = g_{\chi 3} = xg$, $g_{\phi 2} = g_{\psi 3} = g_{\chi 1} = g$, $g_{\phi 3} = g_{\psi 1} = g_{\chi 2} = yg$, $x, y < 1$. (For B_ϕ, the preferential order goes R_1, R_2, R_3, in the case of B_ψ it's R_2, R_3, R_1, and B_χ would have it as R_3, R_1, R_2.) The microbes thus have the same set of growth rates: xg on their first choice, g (the maximum) on the second and yg on their third. Introduce for the sake of convenience

$$\tau_1 = \frac{1}{g}\log\frac{D+2}{3},\ \tau_2 = \frac{1}{g}\log\frac{2D+1}{D+2},\ \tau_3 = \frac{1}{g}\log\frac{3D}{2D+1}, \tag{S.40}$$

so that in an environment shaped by, say, B_ϕ the depletion times will be

$$\Delta T_1 = T_1 = \frac{\tau_1}{x},\ \Delta T_2 = T_2 - T_1 = \tau_2,\ \Delta T_3 = T_3 - T_2 = \frac{\tau_3}{y}. \tag{S.41}$$

Any of the other two anomalous competitors would, on its own, shape the environment in a way identical to the above up to cyclic permutation. Find the values of $x, y < 1$ such that B_ψ (the consummation order R_2, R_3, R_1) can supplant B_ϕ (c. o. R_1, R_2, R_3) upon invasion while B_χ (c. o. R_3, R_1, R_2) would not be able to invade. B_ψ being able to invade means $g_{\psi 2}T_2 + g_{\psi 3}(T_3 - T_2) > \log D$, i. e.

$$xg\,(\frac{\tau_1}{x} + \tau_2) + g\,\frac{\tau_3}{y} > \log D = g\,(\tau_1 + \tau_2 + \tau_3) \tag{S.42}$$

From the above we get

$$\frac{1-y}{y\,(1-x)} > \frac{\tau_2}{\tau_3} \simeq 1.7 \tag{S.43}$$

(the last evaluation we get assuming $D \gg 1$, so that $g\,\tau_2 \simeq \log 2,\ g\,\tau_3 \simeq \log 1.5$ as it is evident from (S.40)). Now, to enable the rock-paper-scissors process, one needs to ensure that B_χ cannot invade the environment shaped by B_ϕ (while B_ψ can if the condition (S.43) holds, and

does chase B_ϕ out upon invasion).

$$g_{\chi 3}\, T_3 = x\, g\, (\frac{\tau_1}{x} + \tau_2 + \frac{\tau_3}{y}) < \log\, D = g\, (\tau_1 + \tau_2 + \tau_3), \tag{S.44}$$

meaning

$$\frac{x - y}{y\, (1 - x)} < \frac{\tau_2}{\tau_3} \simeq 1.7 \tag{S.45}$$

Combining (S.43) with (S.45) we get the rock-paper-scissor condition for the environment that gets repeatedly invaded by the three species B_ϕ, B_ψ, B_χ:

$$\frac{x - y}{y\, (1 - x)} < \frac{\tau_2}{\tau_3} \simeq 1.7 < \frac{1 - y}{y\, (1 - x)} \tag{S.46}$$

The above gets easily satisfied. Indeed, take $x = 0.7$, $y = 0.5$ and get $4/3 < 1.7 < 10/3$ which is obviously true. This means we have organized a rock-paper-scissor cycle: whenever B_ϕ installs itself as the resident, B_χ trying to invade will not succeed, but B_ψ will. However, having chased out B_ϕ, the winner B_ψ will shape the environment so that, due to the symmetry of the system parameters, it will eventually get chased out in its turn by B_χ, and the latter, upon establishing its residence, will get chased by the B_ϕ; at that point, the cycle gets completed and the story will go on.

The question whether a cycle can be organized by the "pedantically normal" species with the preferential list mirroring the table of ranks of their own growth rates, i. e. those that always prefer a nutrient R_i to the nutrient R_j if they grow faster on R_i, we leave unanswered. Though it is interesting in itself and the answer is probably no, to all the real as well is to our modeling purposes the question is academic. We consider a species normal if its top choice is the nutrient on which it grows faster than on all the other nutrients present in the environment. The definition of normality / anomaly is thus environment-dependent: a microbe that seems perfectly normal when its absolute top-choice is present may behave weirdly when the latter is absent (or vice versa). We are perfectly fine with this dependence as it helps us single out a way in which the evolution pressure might go in a particular environment. When we use our definition, the example above is easily modified to present a case in which normal microbes can form a cycle. Add to the environment a universally loved nutrient like glucose and ascribe to all the microbes from the example above the same growth rate $G > g$ on it, making it their first choice. The consideration goes then very similar to the above, although the numerical criterion changes, but the possibility of the cycle remains.

We do not see cycles (or stochasticity, for that matter) in our simulations because the anomalous species vanish from the community as the latter matures, and thus the Pareto-optimal boundary contains only stable steady state points. For a more detailed explanation see below.

E.2 Why the invasion process ends

Suppose there is an endless cycle or stochastic sequence of the steady states (supplanting each other as a result of a successful invasion) that cannot be broken down to an uninvadable state by a series of out-of-turn invasions. By the right of random chance, we are allowed to optimize it: if a steady state 1 is encountered more than once and the states that replace it in the given endless sequence are multiple: 2, 3, ..., then upon encountering the state 1 we always choose the unique invasion that results in the least overall depletion time of the state replacing 1 at this point. As the number of the steady states is finite, and in the end of the optimization each state is supplanted by a particular one, we turned the sequence into a cycle. It is also safe to assume that all the anomalous species are already banned from the community of survivors.

Our argument is, basically, the following. In the mature community all the depletion times are close to $\log D/G$, where G is a typical growth rate of an expert on its top choice. (Here

we call a species "expert" or "champion" of its favorite resource R_i if it is (a) normal and (b) best-growing on R_i among all the normals that share that same top choice.) The cycle is bound to include some invasion events in which a new resident chases out some of the old ones. Usually the successful invader has a higher growth rate on its top choice than the one that is chased out. That however cannot be always the case as it is impossible to have a cycle with the net growth rate on the top choice increasing at each step. So at some point B_ψ with the top choice R_i supplants B_ϕ that prefers R_j even though $g_{\phi j} < g_{\psi i}$ (here some caveats are needed, and we'll get to them later). A crude approximation would have $T_i \sim T_j \sim T \sim \log D/G$, where T is the overall effective growth time; all differences between depletion times $\sim (\Delta G/G^2)\log D$ (where $\Delta G = G_{max} - G_{min}$, G_{max} being the growth rate of the absolute fastest growing on its own top choice champion (or expert), G_{min} — the growth rate of the slowest one among the champions). We would also introduce $\langle g_\phi \rangle$ and $\langle g_\psi \rangle$ as average, in a sense, growth rates of B_ϕ and B_ψ on their second choices and further on, so that we describe the invasion event as

$$g_{\phi j} T_j + \langle g_\phi \rangle (T - T_j) > g_{\psi i} T_i + \langle g_\psi \rangle (T - T_i). \tag{S.47}$$

Resorting to the crude approximations of the differences between times and growth rates, we transform the above into

$$(\langle g_\phi \rangle - \langle g_\psi \rangle) \frac{\Delta G}{G^2} \log D > \Delta G \, \frac{\log D}{G} \tag{S.48}$$

meaning

$$\langle g_\phi \rangle - \langle g_\psi \rangle > G, \tag{S.49}$$

something that is not likely to happen unless the species B_ϕ is anomalous. There are of course those caveats, one of them being that the overall depletion time, though small, can still happen to be larger than $\log D/G_{min}$ (though the worst that can actually happen to it is being of the order $\log D/G + \log(n/(n-1))/\langle g \rangle$ if the last nutrient to be depleted is not occupied, as all the microbe residents having feasted on their favorite choices join in consuming the last unoccupied nutrient with their total concentration over $(n-1)R$, growing upon it with some average rate $\langle g \rangle$, so that $(n-1)R\exp(\langle g \rangle \Delta T_{last}) \sim nR,$), n being the number of resources present in the environment and ΔT_{last} – the short length of time through which only the last nutrient alone is left in the environment. The other is that the invasion event at which the average residential top-choice growth rate gets diminished can be the invasion of the champion of the last nutrient to be depleted with no resident chased out. Still, if that happens, after that one would have to have yet another invasion diminishing the average top-choice growth rate of the first (n - 1) resources, as that one can not grow forever, and the inequality of the same sort as (S.47) will be valid. Even if the invader B_ψ should actually be very good (on the expert level) at growing on its first two nutrients and not necessarily anomalous, it is still a lot to ask of a chance. By increasing the number of species, the probability to get a top choice on the expert level is made smaller and the probability to bring the consummation of two first choices to the expert level plummets down as a square of the former.

All the above is true of course only for the case of a reasonably balanced nutrient supply, while huge imbalance in the resources initial abundances will be favorable for the anomalous species that are good enough and start with the resource which is scarce and excel in their second choice which is abundant (in this way, they are certain to grow mostly on their second choice). The cycle of the sort discussed in the previous section will be hard to organize in this case as it would need certain symmetry, but some complicated way over the Pareto-optimal boundary through its "percolation points" (unstable coexistence points) may be possible.

Even a balanced environment may have a history of this or that resource getting scarce, and for a while can be occupied by the family of the anomalous species. Within such a family, stochasticity can arise, and increase the effective diversity to the point of breaking the rule of the number of the survivors being less than or equal to the number of resources. After all, that rule is valid for the steady states and not for the effective collection of those.

F On the number of surviving species

F.1 Steady state survivors

The usual rule (related to the competitive exclusion principle) stating that “the number of surviving species is less or equal to the number of resources” is applicable to the steady states in the serial dilution experiments, whenever the external conditions are fixed. As always, varying the resource concentrations and / or the dilution coefficient we move the system over the Pareto-optimal boundary, so that different uninvadable steady state communities may assemble themselves under different external conditions.

F.2 Why the competitive exclusion principle applies

Any steady state is associated with a particular depletion order. When only two nutrients are present in the environment, the Time Plane (T_1, T_2) is divided into two regions: above the diagonal $T_1 = T_2$, with $T_1 < T_2$ and consequently the resource R_1 being depleted first, and below the diagonal, with $T_1 > T_2$ hence the reversed depletion order. In the same way, when there are $n > 2$ resources and one has to consider the $n-$dimensional Time Space instead of the Time Plane, the relevant (with all the time coordinates positive) part of the former gets divided by the hyperplanes $T_i = T_j$ into $n!$ segments, each corresponding to a particular depletion order. As long as the depletion order is decided upon, the condition of survival for any microbe can be written in the form of a linear equation. Suppose, for instance, that the depletion order is $T_1, T_2, ..., T_n$ and the bacterium B_α has the preference list beginning with $T_1, T_4, T_{n-2}, T_n, ...$. In this case, in the particular segment of the Time Space the condition for the microbe's survival will be:

$$g_{\alpha 1}\, T_1 + g_{\alpha 4}\, (T_4 - T_1) + g_{\alpha, n-2}\, (T_{n-2} - T_4) + g_{\alpha\, n}\, (T_n - T_{n-2}) = \log\, D$$

We get the number of the survival conditions equal to the number of competing species from the pool $N \gg n$, while the number of unknown depletion time variables is equal to n. Thus obviously, in the general case, no more than n equations can hold simultaneously. There are additional conditions like positiveness of all solutions, uninvadability of a solution, the question of whether the obtained solution is adequate, meaning that it is located on the real and not on the virtual segment of the Pareto-optimal boundary. (To wit, if we get, say, a “single-microbe steady state” corresponding to B_ω growing all the time on its preferred nutrient R_n because it happened to be depleted last, we know that cannot be a real-life solution, as, if left alone, this microbe will eventually change the depletion order by shaping the environment in accordance with its own preference list.) In any case, only a degeneration in the set of the growth rate parameters can lead to surviving of more than n species at the same time (n being the number of the resources present in the environment).

F.3 Trade-off miraculous diversity boost takes too much to be manifested

One specific case of such a degeneration is that of the “trade-off” well known to the researchers familiar with the MacArthur model. If, so to speak, a species can increase its growth rate on a certain source only by decreasing its growth rate on some other nutrients, so that all the microbes in the pool have the same average growth rate, there is a possibility that an arbitrary number of species can coexist, if the conditions are right. Namely, if, for example, the depletion order is $R_1, R_2, ..., R_n$ and the depletion times go as $T_1 = T_2 - T_1 = T_3 - T_2 = ... = T_n - T_{n_1} = \Delta T$, all the microbes with the growth rates constrained by the trade-off condition *and having the same preferential order* in principle can coexist. If the environment is shaped by B_α so that

$$g_{\alpha 1}\, T_1 + g_{\alpha 2}\, (T_2 - T_1) + ... + g_{\alpha\, n}\, (T_n - T_{n-1}) = (g_{\alpha 1} + g_{\alpha 2} + ... + g_{\alpha\, n})\, \Delta T = \log\, D,$$

the same equality will hold for any of the other bacteria sharing that same preferential order and subject to the same trade-off in their growth rates. When added in a seed amount, an

invader, though not really being able to grow, will not die out. Although such a steady state can be theoretically prepared (for any, arbitrarily chosen in advance, set of initial concentrations of the participating microbes), it would be an exotic situation: first, R_2 should be much more abundant than R_1 and R_3 much more abundant than R_2 and so on, the abundances of the sequent nutrients would have to form something close to a geometric progression. Second, the microbes participating in such a community should have strictly the same preferential order; an invader with the same average growth rate but with different preferences would easily win the game as the trade-off will not present to it much of a constraint. This, by the way, should be the case of concern for the MacArthur model as well: if you have the trade-off averaged over, say, 10 resources and there are only three of them in the environment, then the trade-off will not have the chance to manifest itself and the results you get would look as if there was not any.

F.4 Conclusion: the regular rules of the competitive exclusion apply

To conclude, in this paper we deal with a regular computer-resource model for which the rule of "the number of surviving species being less or equal to the number of resources" is applicable as long as it is a steady state and not some cycle or stochastic process at the end of the system's maturation.

G How different yields affect the outcome of the serial dilution experiment

Throughout the main text we assumed all the yields of all the bacterial species wrt different resources to be equal. This however doesn't have to be the case. The effects of differences in terms of yields between species (and, for a single species, wrt different resources) have been known to include multistability [4] and other indications of growing complexity, like "rock, paper, scissors" cycles or stochasticity [8] in the system. In our model, even assuming all the yields equal, we get the above effects when the anomalous microbes manifest themselves. Yet, as we have shown, in the regular situations the anomalous species tend to die out. Accounting for the difference in yields however brings multistability back in the picture.

Dynamics of the environment containing one resource R_1 and one colonizing bacterium B_α within any dilution cycle now reads as

$$\frac{dN_\alpha}{dt} = g_{\alpha 1} N_\alpha\,; \quad \frac{dR_1}{dt} = -\frac{g_{\alpha 1}\, N_\alpha}{Y_{\alpha 1}}, \tag{S.50}$$

where N_α is the concentration of the microbe B_α and $Y_{\alpha 1}$ is the yield of the microbe B_α with respect to the resource R_1. Eq. (S.50) translates into the conservation law valid through a dilution cycle

$$N_\alpha + Y_{\alpha 1}\, R_1 = \text{const.} \tag{S.51}$$

In a steady state, the concentration of the resident should increase $D-$fold through a dilution cycle (D being the dilution coefficient), so that

$$DN_\alpha = N_\alpha + Y_{\alpha 1} R_1, \tag{S.52}$$

where, in contrast with Eqs. (S.50) and (S.51), N_α denotes the steady state concentration of the microbe resident, meaning the value of the concentration with which B_α opens each dilution cycle.

When there are two sources R_1, R_2 present in the medium, and the top choice of B_α is, say, R_1, we get $N_\alpha \exp(g_{\alpha 1} T_1) = N_\alpha + Y_{\alpha 1} R_1$, T_1 being the depletion time of R_1 in the steady state, and

$$DN_\alpha = N_\alpha + Y_{\alpha 1} R_1 + Y_{\alpha 2} R_2. \tag{S.53}$$

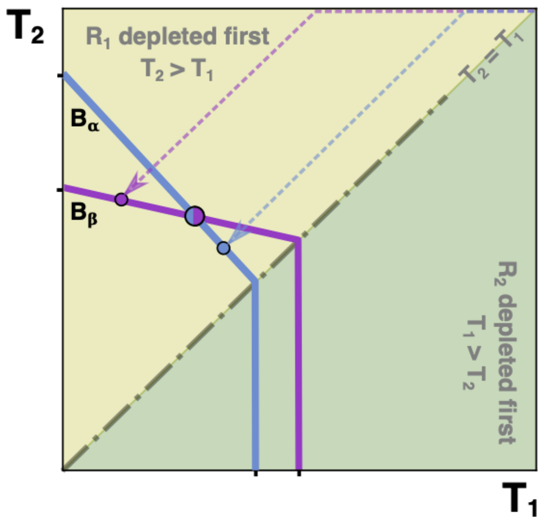

Supplementary Figure 18: **Bistability in the environment of two sources open to invasion by two competing normal microbes.**

Together with the steady state condition in terms of the depletion times of the resources T_1 and T_2 which goes as $g_{\alpha 1} T_1 + g_{\alpha 2} (T_2 - T_1) = \log D$, that defines the depletion times as

$$T_1 = \frac{1}{g_{\alpha 1}} \log \frac{D\, Y_{\alpha 1} R_1 + Y_{\alpha 2} R_2}{Y_{\alpha 1} R_1 + Y_{\alpha 2} R_2}, \quad T_2 = T_1 + \frac{1}{g_{\alpha 2}} \log \frac{D\,(Y_{\alpha 1} R_1 + Y_{\alpha 2} R_2)}{D\, Y_{\alpha 1} R_1 + Y_{\alpha 2} R_2}. \tag{S.54}$$

This is where we have to reconsider the notion that, assuming the balanced nutrient supply, the time spent growing on the top choice is always the largest. Say, if the growth rates are close to each other, $D = 10$, $Y_{\alpha 1} = 0.1$, $Y_{\alpha 2} = 1$, $R_1 = R_2$, then $T_1/T_2 = 0.35\, g_{\alpha 2}/g_{\alpha 1}$, so that T_1 can be considerably less than T_2.

This makes a difference in two ways. First, the anomalous bacteria get a fair chance if they have a small yield on their top choice (unless the dilution coefficient is very large). At this point one can venture a prediction: the anomalous microbes that survive the competition with the normal ones have smaller yields on their top choice. Or, generalizing a statement made in the main text: **the evolutionary pressure encourages the microbes with the diauxie growth to arrange their preferential lists in such a way that either the growth rate on a source gets smaller or the yield gets larger down the list, the lower the rank of the source.** Second, even in the community of the normal microbes multistabilities can occur due to the differences in the yields. It is easily shown that the situation depicted in

Fig. 18 is realizable, when D is not too large, if we allow yields to be different. Whenever the one-microbe line of B_α lands on its ZNGI at the point where B_β cannot invade, and vice versa, the bistability can occur. Indeed, if B_β is the first to invade, in the steady it consumes the resource R_1 favored by both microbes fast enough whenever it has small enough yield wrt R_1, so that B_α cannot invade. If B_α is the first to invade, it grows faster and makes the overall time shorter, so that B_β cannot grow in the environment shaped by its competitor. Hence the steady state is chosen by chance: whoever is the first to invade, wins. All possible complexities can emerge when $D \sim 1/Y$.

We do not withhold the main conclusion concerning the optimization of complementarity even when there is a difference in the yield parameters. The microbe that has the best growth rate on the resource that is the last to disappear always has the advantage and thus can invade. This makes sorting by the top choice inevitable. However, the anomalous microbes might show up and, on rare occasions, become responsible for the low diversity states uninvadable by their normal counterparts.

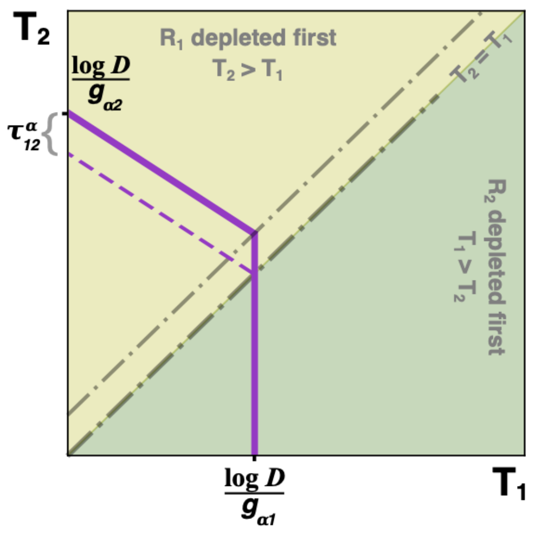

Supplementary Figure 19: **Tilman-inspired diagram for one microbe, the effect of the lag.** The dashed lilac line represents the ZNGI of the microbe B_α with the top choice R_1 in absence of any lag. The solid line of the same color corresponds to the ZNGI with the lag modeled as a time period τ_{12}^{α} of zero growth between the moment of complete consumption of the resource R_1 and that of starting the exponential growth on the resource R_2. There is no distinction between solid and dashed lines when the microbe grows on its top choice only (below the diagonal).

H Taking lags into account

The simplest way to incorporate the lag phase into the model would be to ascribe a special value of the "lag time" to each diauxic shift event. Assume that, sensing that the nutrient currently in use is getting scarce, a microbe pauses in its growth and prepares the metabolic tools for another resource. The pause during which the species B_α switches from R_1 to R_2 we denote as $\tau_{12}^{(\alpha)}$.

The equation defining the microbe's ZNGI would take the following form:

$$g_{\alpha 1} T_1 + g_{\alpha 2}(T_2 - T_1 - \tau_{12}^{(\alpha)}) = \log\, D, \tag{S.55}$$

T_1, T_2 being the depletion times of the resources R_1, R_2; $g_{\alpha 1}$ and $g_{\alpha 2}$ — the growth rates of the species S_α on each on those resources in their turn, D — the dilution coefficient, as usual.

Fig. 20 shows how a lag of the simplest kind is treated by a Tilman-inspired diagram. In the Time plane, the diagonal $T_1 = T_2$ no longer separates the regions corresponding to the two different depletion orders. The boundary shifts up, exactly by the value of the lag time τ_{12}^{α}. The lifeline in the upper region where $T_2 > T_1$ and R_1 is the first to be depleted also shifts up, parallel to the lag-free version, and by the same amount τ_{12}^{α}.

In the main text we have made it clear that, in a mature community, the complementarity tends to become stronger, almost to the point of niche separation, so that (1) depletion times of different resources become close to each other; (2) the feeding strategy of the surviving community in a stable state is, most often, "to each its own top-choice", meaning that the main bulk of the biomass acquired by a species during a dilution step comes from its top-choice nutrient. In fact, the only thing in which the mature community of the species which are "specialists" (meaning that they specialize in using one and only nutrition source) differs from that of the "diauxic shifters", or "switchers" in this particular experimental setting is that, for the latter, the depletion times of the nutrients evolve to become almost equal. This was the conclusion based on the model that does not take lags into account. However, it is even more

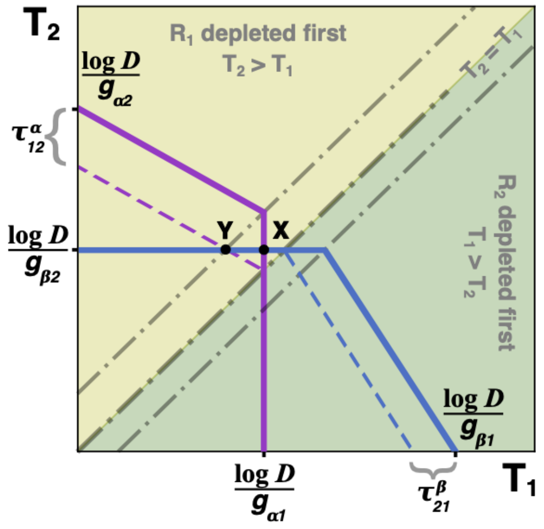

Supplementary Figure 20: **Lags favour specializing.** The dashed segments of the ZNGI lines (lilac, for the microbe B_α and light blue, for B_β) represent the situation in absence of lags. If there had been no lags, both microbes would have been able to coexist at the point **Y**, with B_α having used both resources through a dilution cycle while B_β growing on its top choice R_2. In presence of lags, the coexistence point switches to **X**. At that point, each microbe gets to grow on its own top choice resource only. The lag times are τ_{12}^{α} for B_α (required to switch from the resource R_1 to the resource R_2) and τ_{21}^{β} for B_β (required for switching from the resource R_2 to the resource R_1. Solid lines represent the ZNGI with the lags taken into account.

pronounced when we do take lags into account. It turns out, in a way, lags promote niche separation.

In Fig. 20 the point X indicates the position of coexistence for two species B_α and B_β, lags taken into account. As it is obvious from the way the species' ZNGI lines go, B_α first consumes R_1 and then, after a lag time $\tau_{12}^{(\alpha)}$, switches to the resource R_2, while in the case of the species B_β, the order of preferences is reversed. The point X is where the species coexist, driven to the "neutral band" by their lag times. At this steady state, through each dilution step, each of the microbe grows on its top-choice nutrient only. The lag condition drives the community (consisting, in this particular case, of two species) to a complete (virtual) niche separation. In absence of lags, the two species would have been able to coexist at the point Y, at which B_α consumes R_1, then R_2, while B_β never gets a taste of R_1. If such were the case, the niche separation would have stopped halfway, as the lags had not been there to drive it on.

Regarding the question of a trade-off between the growth rate on a preferred nutrient versus the shortness of the lag phase[9, 10], it does play a role in the community forming.

In absence of such a trade-off, as is seen in Fig. 21, the ZNGI lines of the two species with the same preference order experience a parallel shift and otherwise coexist under the same external conditions (meaning the ratio R_1/R_2). On the other hand, if such a trade-off is present, it may even promote coexistence.

In Fig. 22 the species B_α chooses a large growth rate on its preferred nutrient R_1 at the expense of a huge lag time $\tau_{12}^{(\alpha)}$ while B_β preferring the same nutrient opts for a smaller growth rate while keeping its lag reasonably small as well. With no lag and the same sets of growth rates, the species would not be able to coexist as B_α would win either way. However, as there is that trade-off, they coexist at the point X. (Note that in reality in the absence of that trade-off their growth rates might have been different from those defining the ZNGI slopes in the Fig. 22, so the notion that the trade-off promotes coexistence may be partly misleading.)

The presence of lags makes life harder for the anomalous species whose nutrition strategy is to grow with the higher rate on its second choice rather than on its first. While that may

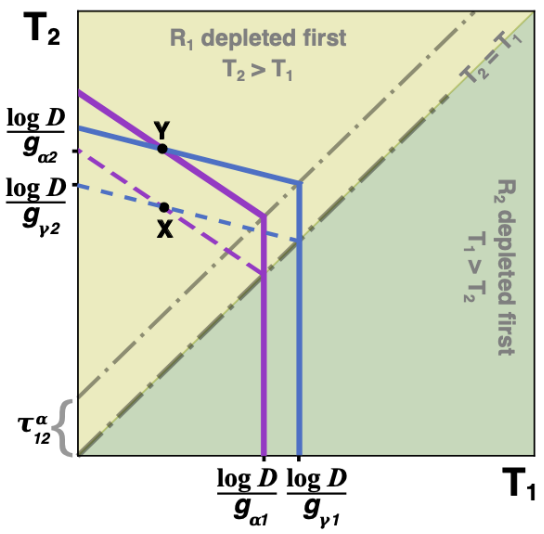

Supplementary Figure 21: **In the absence of a trade-off (lag time vs growth rate).** **X** is the coexistence point of the species B_α and B_γ sharing the same top choice R_1 in the absence of lags. If there is no trade-off between the growth rate and the length of the lag period, accounting for the lag time moves the point **X** up to the point **Y** by parallel translation. The conditions for the coexistence remain the same. (Dashed lines are for ZNGI in absence of the lags, solid lines account for the presence of the lags.)

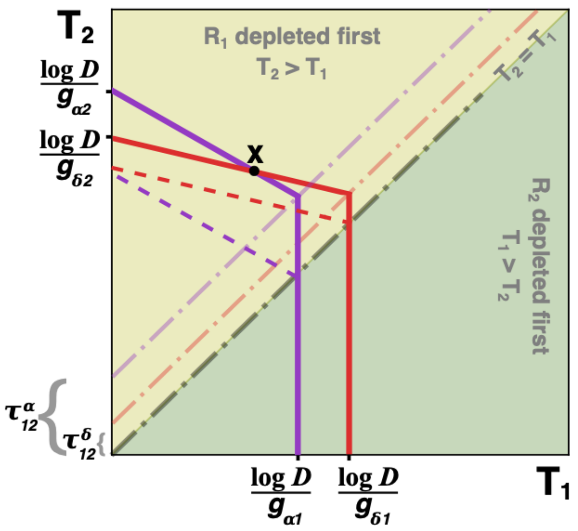

Supplementary Figure 22: **When there is a trade-off (lag time vs growth rate)** In the presence of the trade-off, the bacterium B_α (lilac ZNGI) with the larger growth rate on its top choice R_1 is expected to exhibit longer period of the lag time when switching from R_1 to R_2. At the smaller values of the ratio R_1/R_2 the microbe B_δ (red ZNGI) that opts for the lesser growth rate on its top choice R_1 and shorter lag time gets an advantage. The coexistence is possible at the point **X**. If the microbes had the same growth rates with no lags (or with the lags, but in absence of the trade-off), B_α would have won the competition at any values of the ratio R_1/R_2.

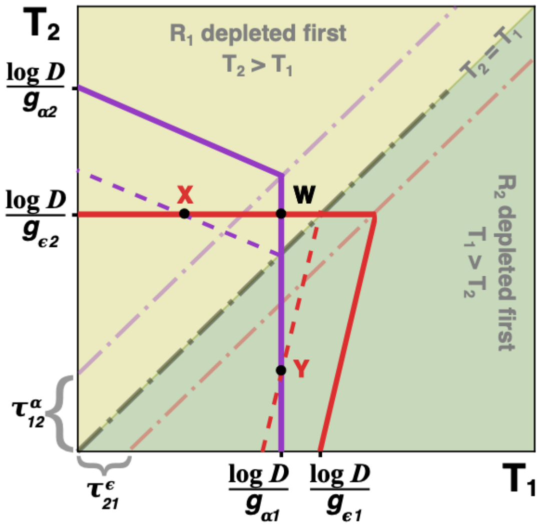

Supplementary Figure 23: **Regular lag times lessen chances of survival for anomalous species.** In absence of lags, the anomalous species B_ϵ (the red ZNGI, dashed "lag-less" version) would have been able to coexist with the normal bacterium B_α (the lilac ZNGI, dashed "lag-less" version) at the point **X** in a stable way and at the point **Y** in an unstable way, allowing the anomalous species become a single uninvadable resident within a particular range of the values of R_1/R_2. The latter option becomes impossible in presence of lags (solid ZNGI lines). The only point of coexistence is now **W** at which the anomalous species does not get to use the resource R_1 at which it grows fastest.

bring success under certain conditions (namely, considerable imbalance in nutrient supply, see the corresponding supplementary section), the advantage is easily lost when (regular) lag times come into light.

In Fig. 23 the red ZNGI corresponds to an anomalous species B_ϵ (that can be judged by the slope of its lifeline at $T_1 > T_2$). In a way, the species is a "superbug": it is not at all bad at competing for its top choice which is R_2, and on its second choice R_1 it grows even faster. The regular lag, however, rends the coexistence point Y ineffective, so that under its most favourable depletion order the species cannot grow. It can still survive at the point W if it is good enough on its top choice, but that would mean it should excel on both accounts while a normal species may be doing fine if it competes reasonably well just for its top choice. This would make the steady states containing anomalous species extremely rare after a considerable number of invasion events. The nature of possible trade-offs however could be finer on that particular point. There are indications[9, 10] that the trade-off goes between the growth rate on the top choice and the length of the lag phase preceding switching from the top choice to the second one. A wild guess would be that in some cases the threshold of "zero lag time" is trespassable, and a reason for some species being anomalous could be that they have, in fact, gone very far in their trade-offs and attained a negative lag time in switching from the nutrient they grow on best to the one on which they grow slower. If this were the case, there would have been no lag in the usual sense between the top choice resource and the second choice for the anomalous species. Can there exist a biological mechanism wild enough to support that wild guess?

In any case, the conclusion of this section is that the features of the mature steady state microbial community (as a result of the modeling done without taking the lag phenomenon into consideration) get even more pronounced due to the presence of lags.

H.1 Multistability induced by lags

Yet another type of multistability that can arise in the serial dilution setting is due to the presence of lags. The lag time is not a solely diauxic feature. If a bacterial culture is starved and is then introduced to a resource, there is usually some time before it can resume growing. Such a regrowth lag is not of a diauxic nature and can be experienced by a co-utilizer [11]. For all we know, its length may be affected by the previous circumstances, i. e. whether the last resource the microbe has grown upon is the same as the one to which it is introduced after a period of starving, or different. In this section we try and take the regrowth lags into account as well as the diauxic lags.

Supplementary Figure 24: **ZNGI with the effects of both regrowth and diauxie lags indicated.** The dashed segments of the ZNGIs stand for the situation of B_α's monoculture in absence of lags. With both lags ($\tau_{12}^{(\alpha)}$ represents the diauxie lag, and $\tau_{21}^{(\alpha)}$ the regrowth lag), the ZNGIs become what is shown by the solid black lines. The steady state is represented by point X.

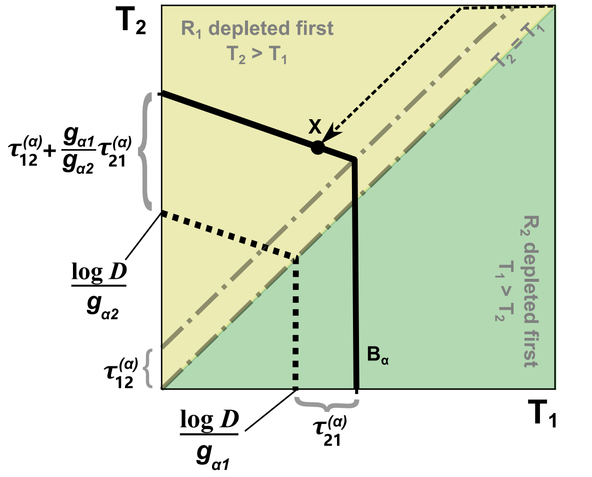

Fig.(24) shows a zero net growth isocline (ZNGI) of a microbe B_α in the Time plane (T_1, T_2): the axes, as usual, being the depletion times of the resources R_1 and R_2 present in the environment. The top choice of the microbe is the resource R_1. When a dilution cycle starts, the (starved) monoculture of the microbe B_α is introduced to the environment containing both R_1 and R_2. The bacterium experiences a regrowth lag $\tau_{21}^{(\alpha)}$ and then starts consuming R_1. Upon depletion of the resource and the diauxic lag period of the length $\tau_{12}^{(\alpha)}$ the microbe switches to the resource R_2, consumes it and is starved for the rest of the dilution cycle. (At the very end, it gets diluted $D-$fold and introduced to the similar environment as at the beginning of the previous cycle.) Note that we use the designation $\tau_{21}^{(\alpha)}$ for the regrowth lag time to indicate that the length of the lag may depend on the history of consumption. The slope of the ZNGI line (where it is inclined wrt both axes) in this model does not depend on the lag values, but the height of the point where it hits the T_2 axis or the degree to which it is raised above the diagonal does. So, in theory, the lag time as a controlling parameter could bring the ZNGI lines of different microbes up or down, make them intersect when they otherwise wouldn't and so on. This, however, could result in a multistability (bistability, if only two sources are available for consumption).

Supplementary Figure 25: **Lags prompting bistability.** With the presence of both regrowth lag and diauxie lag, it is possible that species B_α (ZNGI shown by black solid lines) and B_β (green solid lines) with the same preference order can mutually exclude each other. The monoculture steady states of these two species correspond to point X and Y.

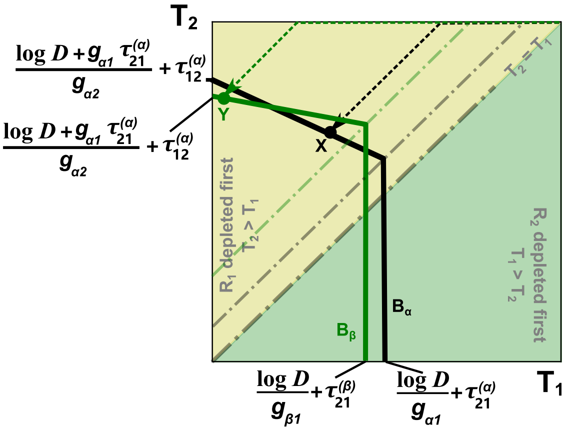

In Fig.(25) the microbe B_α with the steeper slope of the (black) ZNGI has its monoculture steady state situated at the point $(T_1^{(\alpha)}, T_2^{(\alpha)})$ where the microbe B_β (green ZNGI) cannot grow, and vice versa: if B_β is the first to establish its monoculture steady state, B_α would not be able to invade. This can be made possible in two ways. First, if B_α grows faster than B_β on their top choice, i. e. $g_{\alpha 1} > g_{\beta 1}$, its one-microbe line would normally land on its ZNGI to the right on that of B_β (see the subsection **??** of the Supplementary Text). If however the regrowth lag of B_α is longer than that of B_β, i. e. $\tau_{21}^{(\alpha)} > \tau_{21}^{(\beta)}$ (thus moving the former's one microbe line farther to the right), such a disposition is realizable. Second, even if $g_{\alpha 1} < g_{\beta 1}$, but $g_{\alpha 1}/g_{\alpha 2} > g_{\beta 1}/g_{\beta 2}$ and the diauxie lag of B_β is larger than that of B_α, so that $\tau_{12}^{(\alpha)} < \tau_{12}^{(\beta)}$, the intersection may be possible and, as, in this case, the one microbe line of B_α normally lands to the right of that of B_β (if the regrowth lags don't interfere), such a disposition may, again, be realizable.

In Barthe et al. the authors hint that the lag times can actually be controlled in bacteria[1]. If this can be indeed managed, then we are presented with a thrilling possibility to construct complex microbe communities at will, choosing winners or multistable scenarios by manipulating solely the lag parameters.

Now we proceed to deriving a formal criterion that decides the fate of such bistability. We assume $g_{\alpha 1}/g_{\alpha 2} > g_{\beta 1}/g_{\beta 2}$, like it is shown in Fig.25 (the above ratios, or, to be precise, the ratios $(g_{\alpha 1} - g_{\alpha 2})/g_{\alpha 2} > (g_{\beta 1} - g_{\beta 2})/g_{\beta 2}$ controlling the slopes of the upper parts of the ZNGI lines).

The steady state depletion times for the B_α monoculture are

$$\begin{cases} T_1^{(\alpha)} = \dfrac{1}{g_{\alpha 1}} \log\left(\dfrac{D\, R_1 + R_2}{R_1 + R_2}\right) + \tau_{21}^{(\alpha)} \\ T_2^{(\alpha)} = T_1^{(\alpha)} + \tau_{12}^{(\alpha)} + \dfrac{1}{g_{\alpha 2}} \log\left(\dfrac{D\,(R_1 + R_2)}{D\, R_1 + R_2}\right). \end{cases} \tag{S.56}$$

If B_β cannot invade, that means its multiplication coefficient with those depletion times is less than D, i. e.

$$g_{\beta 1}\,(T_1^{(\alpha)} - \tau_{21}^{(\beta)}) + g_{\beta 2}\,(T_2^{(\alpha)} - T_1^{(\alpha)} - \tau_{12}^{(\beta)}) < \log D, \tag{S.57}$$

which, combined with (S.56), gives

$$\frac{g_{\alpha 1}\Big((g_{\beta 2} - g_{\alpha 2})\log D + g_{\alpha 2}\,(g_{\beta 1}\,\Delta\tau_{21} + g_{\beta 2}\,\Delta\tau_{12})\Big)}{g_{\alpha 1}\, g_{\beta 2} - g_{\alpha 2}\, g_{\beta 1}} < \log\left(\frac{DR_1 + R_2}{R_1 + R_2}\right), \tag{S.58}$$

where $\Delta\tau_{21} = \tau_{21}^{(\alpha)} - \tau_{21}^{(\beta)}$ and $\Delta\tau_{12} = \tau_{12}^{(\alpha)} - \tau_{12}^{(\beta)}$.

Note that, for (S.58) to be plausible, the left-hand side of the equation should be less than $\log D$. That implies

$$(g_{\beta 1} - g_{\alpha 1}) \log D + g_{\alpha 1} \left(g_{\beta 1} \Delta\tau_{21} + g_{\beta 2} \Delta\tau_{12}\right) < 0. \tag{S.59}$$

If, on the other hand, the monoculture of B_β was the first to establish itself in the environment, the depletion times of the steady state would be

$$\begin{cases} T_1^{(\beta)} = \dfrac{1}{g_{\beta 1}} \log\left(\dfrac{D\,R_1 + R_2}{R_1 + R_2}\right) + \tau_{21}^{(\beta)} \\ T_2^{(\beta)} = T_1^{(\beta)} + \tau_{12}^{(\beta)} + \dfrac{1}{g_{\beta 2}} \log\left(\dfrac{D\,(R_1 + R_2)}{D\,R_1 + R_2}\right), \end{cases} \tag{S.60}$$

and B_α would not be able to invade if

$$g_{\alpha 1}\,(T_1^{(\beta)} - \tau_{21}^{(\alpha)}) + g_{\alpha 2}\,(T_2^{(\beta)} - T_1^{(\beta)} - \tau_{12}^{(\alpha)}) < \log D, \tag{S.61}$$

which, combined in its turn with (S.60), leads to

$$\frac{g_{\beta 1}\Big((g_{\beta 2} - g_{\alpha 2}) \log D + g_{\beta 2}\,(g_{\alpha 1} \Delta\tau_{21} + g_{\alpha 2} \Delta\tau_{12})\Big)}{g_{\alpha 1}\, g_{\beta 2} - g_{\alpha 2}\, g_{\beta 1}} > \log\left(\frac{DR_1 + R_2}{R_1 + R_2}\right), \tag{S.62}$$

The above can be plausible only if the left-hand side of Eq. (S.62) is positive. That spells as

$$(g_{\beta 2} - g_{\alpha 2}) \log D + g_{\beta 2}\,(g_{\alpha 1} \Delta\tau_{21} + g_{\alpha 2} \Delta\tau_{12}) > 0. \tag{S.63}$$

To be able to match (S.58) with (S.62), one must see to it that the left-hand side of the latter is greater than the left-hand side of the former, and that reduces to

$$-(g_{\alpha 1} - g_{\beta 1})\,(g_{\alpha 2} - g_{\beta 2}) \log D + g_{\alpha 1}\, g_{\beta 1}\,(g_{\alpha 2} - g_{\beta 2})\,\Delta\tau_{21} + g_{\alpha 2}\, g_{\beta 2}\,(g_{\alpha 1} - g_{\beta 1})\,\Delta\tau_{12} < 0. \tag{S.64}$$

The Eqs (S.59), (S.63) and, most importantly, (S.64) form the set of conditions under which there exists a range of the R_1/R_2 ratio values within which bistability can occur. Before we find out the formal range of that ratio in case it exists, let us look closer at the Eq. S.64. First, in absence of lags (or if the corresponding lags are equal to each other, $\tau_{21}^{(\alpha)} = \tau_{21}^{(\beta)}$ and $\tau_{12}^{(\alpha)} = \tau_{12}^{(\beta)}$), the Eqs (S.59), (S.63) and (S.64) are obviously incompatible, so that there may be no bistability in this case. If however it can be assumed that $|\Delta\tau_{12}| \ll |\Delta\tau_{21}|$, (S.64) reduces to

$$(g_{\alpha 2} - g_{\beta 2})(-(g_{\alpha 1} - g_{\beta 1}) \log D + g_{\alpha 1}\, g_{\beta 1}\, \Delta\tau_{21}) < 0 \tag{S.65}$$

If $g_{\alpha 1} > g_{\beta 1}$ and $g_{\alpha 2} < g_{\beta 2}$, then, just as we have discussed in the beginning of this subsection, to get a chance to obtain bistability here we should have $\Delta\tau_{21} > 0$; moreover, as (S.59) in this case actually coincides with (S.65) and (S.63) provides no additional restrictions, we obtain

$$\Delta\tau_{21} > \frac{g_{\alpha 1} - g_{\beta 1}}{g_{\alpha 1}\, g_{\alpha 2}} \log D \tag{S.66}$$

If, on the contrary, the difference in the regrowth lags of both microbes is negligible compared to that of their diauxic lag times, $|\Delta\tau_{12}| \gg |\Delta\tau_{21}|$, then (S.64) becomes

$$(g_{\alpha 1} - g_{\beta 1})(-(g_{\alpha 2} - g_{\beta 2}) \log D + g_{\alpha 2}\, g_{\beta 2}\, \Delta\tau_{12}) < 0 \tag{S.67}$$

If B_α loses to B_β on both accounts in terms of growth rates, $\Delta\tau_{12}$ cannot be positive due to the condition (S.59), and we must have

$$-\frac{g_{\beta 2} - g_{\alpha 2}}{g_{\alpha 2}\, g_{\beta 2}} \log D < \Delta\tau_{12} < -\frac{g_{\beta 1} - g_{\alpha 1}}{g_{\alpha 1}\, g_{\beta 2}} \log D, \tag{S.68}$$

which works out well under our condition $g_{\alpha 1}/g_{\alpha 2} > g_{\beta 1}/g_{\beta 2}$ assumed in the begining for the sake of definitiveness.

Note that, in the case $g_{\alpha 1} > g_{\beta 1}$ Eqs (S.63) and (S.68) become incompatible, so that in this case the bistability can be provided only by manipulating the difference of the regrowth lag times $\Delta\tau_{21}$.

Assume that the conditions (S.59), (S.63) and (S.64) are all satisfied and proceed to determine the range of R_1/R_2 ratio that favours bistability. Introduce the notations

$$x = \frac{g_{\alpha 1}\left(g_{\beta 2} - g_{\alpha 2}\right)}{g_{\alpha 1}\, g_{\beta 2} - g_{\alpha 2}\, g_{\beta 1}}$$
$$y = \frac{g_{\alpha 1}\, g_{\alpha 2}\left(g_{\beta 1}\,\Delta\tau_{21} + g_{\beta 2}\,\Delta\tau_{12}\right)}{g_{\alpha 1}\, g_{\beta 2} - g_{\alpha 2}\, g_{\beta 1}}. \tag{S.69}$$

Eq. (S.58) then takes the form

$$D^x \exp(y) < \frac{D R_1 + R_2}{R_1 + R_2}. \tag{S.70}$$

As we have made sure that $D^x \exp(y) < D$, the above is equivalent to

$$\frac{R_1}{R_2} > \frac{D^x \exp(y) - 1}{D - D^x \exp(y)}. \tag{S.71}$$

Again, introduce the notations:

$$u = \frac{g_{\beta 1}\left(g_{\beta 2} - g_{\alpha 2}\right)}{g_{\alpha 1}\, g_{\beta 2} - g_{\alpha 2}\, g_{\beta 1}}$$
$$v = \frac{g_{\beta 1}\, g_{\beta 2}\left(g_{\alpha 1}\,\Delta\tau_{21} + g_{\alpha 2}\,\Delta\tau_{12}\right)}{g_{\alpha 1}\, g_{\beta 2} - g_{\alpha 2}\, g_{\beta 1}}. \tag{S.72}$$

The left-hand side of Eq. (S.62) will than read as $D^u \exp(v)$. There is a possibility that $D^u \exp(v) > D$, i. e.

$$\left(g_{\beta 1} - g_{\alpha 1}\right)\log D + g_{\beta 1}\left(g_{\alpha 1}\,\Delta\tau_{21} + g_{\alpha 2}\,\Delta\tau_{12}\right) > 0, \tag{S.73}$$

then there are no additional restrictions coming from the fact that g_α cannot invade B_β−monoculture, so that (S.71) defines the region of R_1/R_2 favouring the bistability phenomenon. If however (S.73) does not hold, and the opposite is true, then we get yet another condition

$$D^u \exp(v) > \frac{D R_1 + R_2}{R_1 + R_2}, \tag{S.74}$$

which, combined with (S.71), results in the final bistability condition

$$\frac{D^x \exp(y) - 1}{D - D^x \exp(y)} < \frac{R_1}{R_2} < \frac{D^u \exp(v) - 1}{D - D^u \exp(v)}, \tag{S.75}$$

with x, y given by (S.69) and u, v by (S.74).

To conclude, we note that if, say, $g_{\alpha 1} > g_{\beta 1}$ and $g_{\alpha 2} < g_{\beta 2}$, the lag times and the dilution coefficient can be arranged so that the competition between the two species would rend any of them as a sole winner, or result in their co-existence, or even display the tendency towards bistability.

I Representing the co-utilizing microbes in the time plane

Earlier (in the main text) we have claimed that the geometrical approach we have developed is useful whichever metabolic strategy is chosen by the microbes in the environment subject to

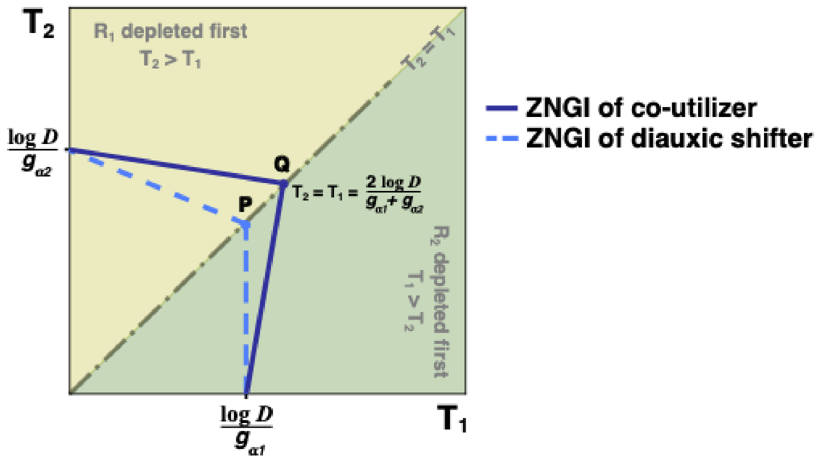

Supplementary Figure 26: **Zero net growth isoclines: co-utiliser vs diauxic shifter in the Time plane.** The co-utilising microbe (violet ZNGI) and the diauxie shifting microbe (dashed blue ZNGI, top choice R_1) technically have the same growth rates on both resources R_1 and R_2. However, in absence of lags the diauxie shifter's ZNGI is located inside the Time plane segment bounded by the co-utiliser's zero net growth isocline, so that at any values of the ratio R_1/R_2 the former would win the competition. One of the key indicators of how a microbe fares at a roughly balanced nutrient supply is the position of the point at which the microbe's ZNGI intersects with the diagonal $T_1 = T_2$. It can be noted that the point P associated with the diauxie-shifting microbe is closer to the origin than the point Q associated with the co-utiliser.

serial dilution. Here we suggest the way to treat the co-utilizing microbes in the context of the serial dilution set-up. As in the case with the metabolic strategy involving diauxie, we assume that each dilution cycle starts with the initial abundance of resources sufficiently high to ensure exponential growth of the resident microbes. We also assume for simplicity that the co-utilising microbes given n resources allocate to each one $1/n$ fraction if its metabolic engines, so that a microbe B_α grows on the resources $R_1, R_2, ..., R_n$ with the rate $(g_{\alpha 1} + g_{\alpha_2} + ... + g_{\alpha n})/n$. That would mean that, in the case of the environment containing two resources R_1, R_2, the microbe B_α that happens to deplete first R_1 and then R_2 (utilising both while they are available), for the time period T_1 will grow with the rate $(g_{\alpha 1} + g_{\alpha 2})/2$ and then for the time period $T_2 - T_1$ with the rate $g_{\alpha 2}$. Hence the zero net-growth isocline (ZNGI) for this particular depletion order will have the equation

$$\frac{g_{\alpha 1} + g_{\alpha 2}}{2} T_1 + g_{\alpha 2} (T_2 - T_1) = \log D, \tag{S.76}$$

where D is, as usual, the dilution coefficient. Should the depletion order happen to be reversed, so that the depletion times become such that $T_1 > T_2$, B_α would then grow first for the time period T_2 with the rate $(g_{\alpha 1} + g_{\alpha 2})/2$ and then, for the time period $T_1 - T_2$, with the rate $g_{\alpha 1}$. That depletion order corresponds to the part of the ZNGI having the equation

$$\frac{g_{\alpha 1} + g_{\alpha 2}}{2} T_2 + g_{\alpha 1} (T_1 - T_2) = \log D. \tag{S.77}$$

When both resources are depleted at the same time, one gets $T_1 = T_2 = \log D/((g_{\alpha 1} + g_{\alpha 2})/2)$, which is different from the case of the diauxic shifters. For the latter, when the depletion times are equal, they are also equal to $\log D/\max(g_1, g_2)$, i. e. (for the normal species) with the maximum of the two growth rates on two different nutrients in the denominator rather than the average growth rate. This difference implies that, in the case of the co-utilizing microbes, the growth rate "on the top choice" (which translates simply into the largest growth rate, as the co-utilizing microbes use all the resources simultaneously) may cease to be the main selection parameter.

In Fig. 26 we have shown the ZNGI line for the co-utiliser B_α (solid line) and (dashed line) the way the same isocline would go if B_α were a diauxic shifter, with the same values of growth rates, and the preference order first R_1, then R_2. The figure may be misleading in two ways: (1) the co-utilisers metabolic strategy is modeled too crudely; (2) in the case of the diauxic shifter, it does not show any lag. Still, the message is clear: as long as the lag is smaller than some critical lag value determined by the growth rates and the dilution rate, in the case of the balanced (and regular) nutrient supply it is more reasonable to opt for a diauxic shift than for co-utilising.

We do not go into further details concerning the (fascinating) differences between the two metabolic strategies (easily brought to light by the graphic approach), as those are clearly beyond the scope of this particular work.

J Coexistence of the diauxic species having the same preferential orders

While the species with the same preferential order with regard to the carbon sources are naturally pressed harder by the competitive exclusion principle, they can still co-exist when the external conditions are right. The advantage of our model being its simplicity, the set of the external conditions in our case boils down to that of the relative abundances of the initial resource concentrations, e. g. R_1/R_2 if there are two resources available, and the value of the dilution coefficient D. In this subsection we will obtain the conditions allowing for the coexistence of two species B_α and B_β, both having the resource R_1 as their top choice, and, upon its depletion, switching to the resource R_2 in a diauxie manner. We will also try to include the diauxic lags into the consideration as well as point out the conditions under which their presence may be neglected. Using the data provided by Barthe et al. [1], we will make a testable prediction specifying, for various values of the dilution coefficient D, the region of R_1/R_2 favourable for the coexistence of the strains $E22$ and $M1/5$ of the bacterium *E. coli* growing on a mixture of glucose and xylose in the serial dilution setting.

Consider microbes B_α and B_β able to grow on the carbon sources R_1 and R_2 in a diauxic manner with the growth rates $g_{\alpha 1} > g_{\beta 1}$ on the first resource and $g_{\alpha 2} < g_{\beta 2}$ on the second. When R_1 and R_2 are both available, the microbes start with R_1 and, when it is consumed, after the lag periods $\tau_{12}^{(\alpha)}$ and $\tau_{12}^{(\beta)}$ respectively start consuming R_2. Presumably at the beginning of the next dilution cycle both microbes demonstrate a regrowth lag when being intoduced again to R_1, $\tau_{21}^{(\alpha)}$ and $\tau_{21}^{(\beta)}$ respectively. If the microbes share the environment, coexisting in a steady state, then their intial concentrations get multiplied by the factor D till the end of each dilution cycle. Due to the exponential nature of their growth, we get the equations

$$\begin{cases} g_{\alpha 1}\,(T_1 - \tau_{21}^{(\alpha)}) + g_{\alpha 2}\,(T_2 - T_1 - \tau_{12}^{(\alpha)}) = \log D \\ g_{\beta 1}\,(T_1 - \tau_{21}^{(\beta)}) + g_{\alpha 2}\,(T_2 - T_1 - \tau_{12}^{(\beta)}) = \log D, \end{cases} \tag{S.78}$$

where T_1 and T_2 are the depletion times of the resources R_1 and R_2 respectively, as usual. We get

$$T_1 = \frac{(g_{\beta 2} - g_{\alpha 2})\,\log D + g_{\beta 2}\,g_{\alpha 1}\,\tau_{21}^{(\alpha)} - g_{\alpha 2}\,g_{\beta 1}\,\tau_{21}^{(\beta)} + g_{\alpha 2}\,g_{\beta 2}(\tau_{12}^{(\alpha)} - \tau_{12}^{(\beta)})}{g_{\alpha 1}\,g_{\beta 2} - g_{\alpha 2}\,g_{\beta 1}} \tag{S.79}$$

and

$$T_2 = \frac{1}{g_{\alpha 1}\,g_{\beta 2} - g_{\alpha 2}\,g_{\beta 1}}\Big((g_{\alpha 1} - g_{\alpha 2} - g_{\beta 1} + g_{\beta 2})\,\log D + \\ + (g_{\alpha 1} - g_{\alpha 2})\,(g_{\beta 1}\,\tau_{21}^{(\beta)} + g_{\beta 2}\,\tau_{12}^{(\beta)}) - (g_{\beta 1} - g_{\beta 2})\,(g_{\alpha 1}\,\tau_{21}^{(\alpha)} + g_{\alpha 2}\,\tau_{12}^{(\alpha)})\Big) \tag{S.80}$$

In order to obtain the coexistence conditions for B_α and B_β we determine in a formal way the steady state initial concentrations N_α, N_β of the microbes assuming they share the environment and then find at which values of the ratio R_1/R_2 both N_α and N_β thus obtained are positive. (The alternative approach is to use the Tilman-inspired diagrams, and we will discuss it later in that same subsection as well.)

Denote $\Delta T_1^{(\alpha)} = T_1 - \tau_{21}^{(\alpha)}$ and $\Delta T_1^{(\beta)} = T_1 - \tau_{21}^{(\beta)}$ — the time periods during which B_α and B_β respectfully grow on R_1 until it is finally depleted. Then, at the moment when the first resource is consumed, the biomass conservation law would have it

$$N_\alpha \exp\left(g_{\alpha 1}\,\Delta T_1^{(\alpha)}\right) + N_\beta \exp\left(g_{\beta 1}\,\Delta T_1^{(\beta)}\right) = R_1 + N_\alpha + N_\beta. \tag{S.81}$$

The steady state condition (or the same conservation law at the point when both resources are consumed), on the other hand, requires

$$D\,N_\alpha + D\,N_\beta = R_1 + R_2 + N_\alpha + N_\beta. \tag{S.82}$$

Combining (S.81) and (S.82) we get

$$\begin{cases} N_\alpha = \dfrac{\Big(D - \exp\left(g_{\beta 1}\,\Delta T_1^{(\beta)}\right)\Big)\,R_1 - \Big(\exp\left(g_{\beta 1}\,\Delta T_1^{(\beta)}\right) - 1\Big)\,R_2}{(D-1)\Big(\exp\left(g_{\alpha 1}\,\Delta T_1^{(\alpha)}\right) - \exp\left(g_{\beta 1}\,\Delta T_1^{(\beta)}\right)\Big)} \\[2ex] N_\beta = \dfrac{\Big(\exp\left(g_{\alpha 1}\,\Delta T_1^{(\alpha)}\right) - 1\Big)\,R_2 - \Big(D - \exp\left(g_{\alpha 1}\,\Delta T_1^{(\alpha)}\right)\Big)\,R_1}{(D-1)\Big(\exp\left(g_{\alpha 1}\,\Delta T_1^{(\alpha)}\right) - \exp\left(g_{\beta 1}\,\Delta T_1^{(\beta)}\right)\Big)} \end{cases} \tag{S.83}$$

It is required that $N_\alpha > 0$, $N_\beta > 0$, so that the region of the values of the ratio R_1/R_2 favorable to the coexistence of both species is restricted to

$$\frac{\exp\left(g_{\beta 1}\,\Delta T_1^{(\beta)}\right) - 1}{D - \exp\left(g_{\beta 1}\,\Delta T_1^{(\beta)}\right)} < \frac{R_1}{R_2} < \frac{\exp\left(g_{\alpha 1}\,\Delta T_1^{(\alpha)}\right) - 1}{D - \exp\left(g_{\alpha 1}\,\Delta T_1^{(\alpha)}\right)} \tag{S.84}$$

The explicit expressions for $\Delta T_1^{(\alpha)} = T_1 - \tau_{21}^{(\alpha)}$ and $\Delta T_1^{(\beta)} = T_1 - \tau_{21}^{(\beta)}$ are

$$\Delta T_1^{(\alpha)} = \frac{(g_{\beta 2} - g_{\alpha 2})\,\log D + g_{\beta 1}\,g_{\alpha 2}\,(\tau_{21}^{(\alpha)} - \tau_{21}^{(\beta)}) + g_{\alpha 2}\,g_{\beta 2}\,(\tau_{12}^{(\alpha)} - \tau(\beta)_{12})}{g_{\alpha 1}\,g_{\beta 2} - g_{\alpha 2}\,g_{\beta 1}} \tag{S.85}$$

and

$$\Delta T_1^{(\beta)} = \frac{(g_{\beta 2} - g_{\alpha 2})\,\log D + g_{\beta 2}\,g_{\alpha 1}\,(\tau_{21}^{(\alpha)} - \tau_{21}^{(\beta)}) + g_{\alpha 2}\,g_{\beta 2}\,(\tau_{12}^{(\alpha)} - \tau(\beta)_{12})}{g_{\alpha 1}\,g_{\beta 2} - g_{\alpha 2}\,g_{\beta 1}} \tag{S.86}$$

From (S.85) and (S.86) it is obvious that when the difference in the lag times for the two microbes is small enough (or, in the other words, the dilution factor D is large enough), so that

$$\log D \gg \frac{g_{\beta 2}\,g_{\alpha 1}\,\Delta\tau_{21} + g_{\alpha 2}\,g_{\beta 2}\Delta\tau_{12}}{g_{\beta 2} - g_{\alpha 2}} \tag{S.87}$$

($\Delta\tau_{21}$ and $\Delta\tau_{12}$ here being the differences in the corresponding lags for two microbes), then the lags in (S.84) can be neglected.

For two strains of Escherichia coli, $B_\alpha = M1/5$ and $B_\beta = E22$, the growth rates on $R_1 =$ glucose and $R_2 =$ xylose given by [1] are $g_{\alpha 1} = 0.9h^{-1}$, $g_{\alpha 2} = 0.35h^{-1}$ and $g_{\beta 1} = 0.75h^{-1}$, $g_{\beta 2} = 0.65h^{-1}$ respectively. Even if the relevant values of $\Delta\tau$ were of the order of 0.5 hour, we would have the right hand side of the Eq. (S.87) $\sim 0.4 - -0.5$, which is about one tenth of $\log D$ at $D = 100$. Judging by the data exposed by [1] however the lag times themselves are

less or equal to 0.5 hour, so that the difference between them must be even less. This is where we can hope to lose nothing much by neglecting the terms associated with the lag times τ in the nominator of (S.84). Bearing this in mind, we transform (S.84) into

$$\frac{D^x - 1}{D - D^x} < \frac{R_1}{R_2} < \frac{D^y - 1}{D - D^y} \tag{S.88}$$

with

$$x = \frac{g_{\beta 1}\,(g_{\beta 2} - g_{\alpha 2})}{g_{\alpha 1} g_{\beta 2} - g_{\beta 1} g_{\alpha 2}} \text{ and } y = \frac{g_{\alpha 1}\,(g_{\beta 2} - g_{\alpha 2})}{g_{\alpha 1} g_{\beta 2} - g_{\beta 1} g_{\alpha 2}}.$$

The condition (S.88) is relevant whenever the corresponding lag times of the co-existing microbes are close enough to each other, as they tend to mutually cancel each other. In our particular case, with B_α and B_β being the $M1/5$ and $E22$ strains of E-coli, this boils down to

$$\frac{D^{0.70} - 1}{D - D^{0.70}} < \frac{R_1}{R_2} < \frac{D^{0.84} - 1}{D - D^{0.84}} \tag{S.89}$$

To make our message more practical, we calculate the range of the ratio of the glucose and xylose concentrations favorable for the coexistence of the two *E. coli* strains ($B_\alpha = M1/5$ an $B_\beta = E22$) for the values of the dilution factor $D = 10, 100, 1000$ *and* 10000. The ranges are:

- for $\mathbf{D} = \mathbf{10}$ it's $0.80 < \frac{R_1}{R_2} < 1.92$;
- for $\mathbf{D} = \mathbf{100}$ it's $0.32 < \frac{R_1}{R_2} < 0.90$;
- for $\mathbf{D} = \mathbf{1000}$ it's $0.14 < \frac{R_1}{R_2} < 0.49$;
- for $\mathbf{D} = \mathbf{10000}$ it's $0.07 < \frac{R_1}{R_2} < 0.19$.

With the increase of the dilution factor D, the interval of coexistence gets shorter as the number of generations needed to obtain the fitting multiplication factor becomes larger and the differences tend to accumulate with generations.

The other way to derive the conditions (S.84) and (S.88) is to consider the diagram showing the Zero Net Growth isoclines of both microbes in the time plane (the coordinate axes being the depletion times of the carbon sources), in the spirit of Tilman's mechanistic approach. The one-microbe lines of the species (i. e. the dynamic trajectories of the monoculture states) land on the isoclines at the points corresponding to the steady states of the monocultures. The depletion times $T_1^{(\alpha)}$ and $T_1^{(\beta)}$ pertaining to the steady state of the monocultures B_α and B_β can be obtained in the same way as described above, from the biomass conservation laws: namely, when the first resource is depleted, one writes $N_\alpha \exp\left(g_{\alpha 1}(T_1^{(\alpha)} - \tau_\alpha^{21})\right) = R_1 + N_\alpha$ (for the monoculture of the microbe B_α) and $N_\beta \exp\left(g_{\beta 1}(T_1^{(\beta)} - \tau_\beta^{21})\right) = R_1 + N_\beta$ (for the monoculture of the microbe B_β). Coupled with the biomass conservation law applied at the point when both resources are consumed and the steady state condition requiring that a microbe should multiply its population by the factor D at the end of the dilution cycle, $D\,N_\alpha = R_1 + R_2 + N_\alpha$ (or $D\,N_\beta = R_1 + R_2 + N_\beta$, respectfully), one obtains the monoculture depletion times as

$$T_1^{(\alpha)} = \frac{1}{g_{\alpha 1}} \log\left(\frac{DR_1 + R_2}{R_1 + R_2}\right) + \tau_\alpha^{21} \tag{S.90}$$

and

$$T_1^{(\beta)} = \frac{1}{g_{\beta 1}} \log\left(\frac{DR_1 + R_2}{R_1 + R_2}\right) + \tau_\beta^{21} \tag{S.91}$$

Supplementary Figure 27: **Coexistence of the diauxic species having the same preferential orders.** The two *E. coli* species, with their non-zero initial and regrowth lags represented by τ's, can coexist despite having the same preference order on the two supplied resources. The monoculture steady state of species α (purple ZNGI) corresponds to the purple point, a state where species β (red ZNGI) can successfully invade, and vice versa. The steady state of coexistence is at the black point X, where both ZNGIs intersect.

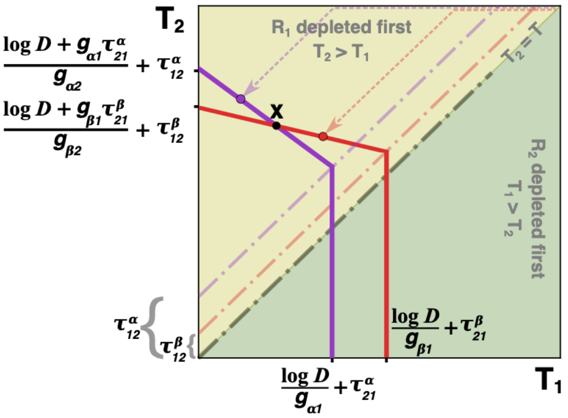

In Fig. 27 one can see that when the environment is shaped by the monoculture B_α is invadable by B_β if the depletion time $T_1^{(\alpha)}$ is less than that required for coexistence. On the other hand, when the monoculture B_β shapes the environment, the microbe B_α can invade if the depletion time $T_1^{(\beta)}$ is greater than that of coexistence. We get

$$T_1^{(\alpha)} < \frac{(g_{\beta 2} - g_{\alpha 2})\,\log D + g_{\beta 2}\, g_{\alpha 1}\, \tau_{21}^{(\alpha)} - g_{\alpha 2}\, g_{\beta 1}\, \tau_{21}^{(\beta)} + g_{\alpha 2}\, g_{\beta 2}(\tau_{12}^{(\alpha)} - \tau_{12}^{(\beta)})}{g_{\alpha 1}\, g_{\beta 2} - g_{\alpha 2}\, g_{\beta 1}} < T_1^{(\beta)} \qquad \text{(S.92)}$$

From this, (S.84) and (S.88) are easily obtained. Thus here, as usual, one can use either diagrams or more formal approach to derive the criterion.

K What happens to complementarity and diversity when the growth rate distributions are not similar for different sources

Through the main text we have assumed that the nutritional value of different resources is the same, meaning that the growth rate distributions of the different microbes with respect to different resources are similar to each other. Obviously, if we relax this assumption to a large extent, the conclusions concerning both diversity and complementarity of the mature state community of survivors will require modifications. Yet another conclusion that would have to be revisited in this case is that of the eventual extinction of the anomalous microbes. The lag times that are great equalizers will become more important when the growth rates on different sources can be very different. In this subsection, we will address both diversity and complementarity concerns and then discuss in which way a microbe may benefit from adopting an anomalous metabolic strategy when the distributions of the growth rates on different sources that are available in the environment are not equal.

Suppose there is a universally prized resource on which all the microbes grow comparatively fast, like glucose, and it is present in the environment. We could reasonably expect the distributions of the growth rates on such a resource to have a higher mean value and probably smaller variance than those for a less popular resource. In Fig. 28 a Tilman-inspired diagram (Time plane) shows in which way the difference in the growth rates distributions on two resources R_1 and R_2 affects the community of three microbes B_α (top choice R_1, blue ZNGI), B_β (top choice R_1, orange ZNGI) and B_γ (top choice R_2, magenta ZNGI). Due to the fact that the growth

Supplementary Figure 28: **Complementarity broken by the dissimilarity of the growth rates distributions on two sources.** The ZNGI lines of the experts on R_1 (the microbe B_α, top choice R_1, blue ZNGI) and on R_2 (the microbe B_γ, top choice R_2, magenta ZNGI) intersect at the point **Z**, which is driven out of the Pareto-optimal boundary by the segment **XY** of the ZNGI line of a microbe B_β (top choice R_1, orange ZNGI). Thus the experts cannot co-exist in presence of B_β. The intersection point **X** of the ZNGI of B_β (orange) and that of B_γ (magenta) can be realized at small values of $R_1/R_2 \ll 1$. The intersection point **Y** of the ZNGI of B_α (blue) and that of B_β (orange) may or may not be realized as a co-existence steady state at a balanced resource supply $R_1/R_2 = 1$, depending on the set of parameters (growth rates, dilution coefficient) of the system.

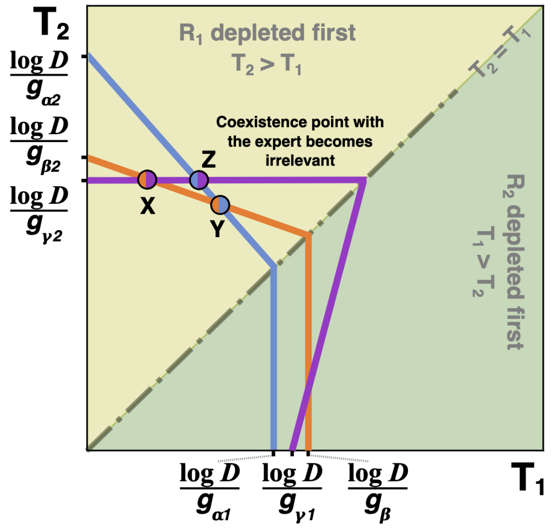

rates on R_1 are greater than those on R_2 any microbe with the top choice R_2 (like B_γ in our case) will be anomalous by definition. The segment of the Pareto-optimal boundary that allows for the presence of B_γ in the resulting uninvadable steady state joins a point at the T_2 axis to the point **X**. This means that B_γ may be present in the community (always in co-existence with B_β) only at a very imbalanced nutrient supply, with $R_1/R_2 \ll 1$. This is where we may still have complementarity. The "community of experts" that we have often encountered in our simulations through the main text of the current paper cannot be the resulting set of survivors at the uninvadable steady state. The point **Z** that corresponds to such a situation does not appear on the Pareto-optimal boundary.

K.1 The criterion for the presence of the complementarity, lags neglected

Now we proceed to derive a criterion deciding the fate of complementarity at the balanced resource supply in the case of two resources available in the medium, R_1 being the one that provides the fastest growth for all (or most) microbes. Let B_γ be an expert on R_2. It is reasonable to assume that the depletion order in the case under consideration is such that R_1 is consumed first. Under this assumption, the only microbe with the top choice R_2 that has a chance to survive is the expert – the one that grows on R_2 faster than any other microbe. (If such a microbe prefers R_1 to R_2, there can be no complementarity with that depletion order, as in this case the expert on R_2, through a dilution cycle, will use both resources and grow on each of them faster than any other microbe would on R_2 only.) Let B_β be the microbe whose ZNGI is the first to intersect with the ZNGI of B_γ on the left, with the intersection point closest to T_2 axis. Coexistence between them is possible if B_γ can grow at the point at which B_β monoculture is established.

In the steady state of B_β monoculture the depletion times are

$$T_1^{(\beta)} = \frac{1}{g_{\beta 1}} \log\left(\frac{D\,R_1 + R_2}{R_1 + R_2}\right),$$
$$T_2^{(\beta)} = T_1^{(\beta)} + \frac{1}{g_{\beta 2}} \log\left(\frac{D\,(R_1 + R_2)}{D\,R_1 + R_2}\right), \tag{S.93}$$

so that, for B_γ to be able to grow, we must have

$$g_{\gamma 2}\left[\frac{1}{g_{\beta 1}} \log\left(\frac{D\,R_1 + R_2}{R_1 + R_2}\right) + \frac{1}{g_{\beta 2}} \log\left(\frac{D\,(R_1 + R_2)}{D\,R_1 + R_2}\right)\right] > \log D. \tag{S.94}$$

The above reduces to

$$\frac{g_{\beta 1}\,(g_{\gamma 2} - g_{\beta 2})}{g_{\gamma 2}\,(g_{\beta 1} - g_{\beta 2}} \log D > \log\left(\frac{D\,R_1 + R_2}{R_1 + R_2}\right). \tag{S.95}$$

Introducing a notation

$$x = \frac{g_{\beta 1}\,(g_{\gamma 2} - g_{\beta 2})}{g_{\gamma 2}\,(g_{\beta 1} - g_{\beta 2})}, \tag{S.96}$$

we get from (S.95):

$$\frac{R_1}{R_2} < \frac{D^x - 1}{D - D^x}. \tag{S.97}$$

As we are especially interested in the case with the balanced resource supply $R_1 = R_2$, we get

$$\frac{D + 1}{2} < D^x, \tag{S.98}$$

which, with $D \gg 1$, reduces to

$$\boxed{\frac{g_{\beta 2}\,(g_{\beta 1} - g_{\gamma 2})}{g_{\gamma 2}\,(g_{\beta 1} - g_{\beta 2})} < \frac{1}{\log_2 D}} \tag{S.99}$$

When $g_{\beta 1} \gg g_{\gamma 2}$ that would mean huge dispersion for the growth rate distribution on the resource R_2, as (S.99) in this case reduces to simply

$$\frac{g_{\beta 2}}{g_{\gamma 2}} < \frac{1}{\log_2 D} \tag{S.100}$$

for a microbe B_β with some average (or even greater than average) growth rate on R_2.

If however

$$g_{\alpha 1} - g_{\gamma 2} < \sigma_2 \frac{1}{\log_2 D}, \tag{S.101}$$

where $B_{\alpha 1}$ is the expert on R_2 and σ_2 is the dispersion of the growth rate distribution on R_2, then for all practical purposes we can have complementarity at the balanced resource supply, as in (S.99) $g_{\beta 1} - g_{\gamma 2} < g_{\alpha 1} - g_{\gamma 2}$ and we could reasonably expect $g_{\beta 1} - g_{\beta 2} = g_{\beta 1} - g_{\gamma 2} + g_{\gamma_2} - g_{\beta 2} \simeq g_{\beta 1} - g_{\gamma 2} + \sigma_2$ to exceed the dispersion σ_2.

To be more precise we should put in the right side of (S.101) the mathematical expectations of the maximum values of the growhth rates on the corresponding sources, i. e. **the official practical complementarity criterion for the balanced nutrient supply** should be formulated in the following way:

$$\boxed{g_{1max} - g_{2max} < \sigma_2 \frac{1}{\log_2 D}}. \tag{S.102}$$

We should also note that (S.98) (or its detailed version in Eq. (S.99)) must hold for any microbe with the top choice R_2, otherwise the complementary coexistence of B_γ and B_β will be invadable.

K.2 When the diauxic lags enter the picture: complementarity preserved and the anomalous microbes getting their chance

Assume that the microbe B_β with the top choice R_1 experiences have a diauxic lag period τ_{12}^{β} when the resource R_1 is depleted, before switching to consumption of the resource R_2. (Let the regrowth lags of all the microbes be approximately equal to each other: the differences may play their role either to disrupt or strengthen the complementarity positions, but the diauxic lag is more important for the reason that for the bacteria with the top choice R_1 it is manifested while for those with the top choice R_2 it does not occur when the depletion order is first R_1 then R_2.) Then Eq. (S.93) should be modified so that the depletion time T_2 in the steady state occupied by B_β alone is

$$T_2^{(\beta)} = T_1^{(\beta)} + \tau_{12}^{(\beta)} + \frac{1}{g_{\beta 2}} \log\left(\frac{D\,(R_1 + R_2)}{D\,R_1 + R_2}\right), \tag{S.103}$$

so that B_γ with the top choice R_2 can invade if

$$g_{\gamma 2}\left[\frac{1}{g_{\beta 1}} \log\left(\frac{D\,R_1 + R_2}{R_1 + R_2}\right) + \tau_{12}^{(\beta)} + \frac{1}{g_{\beta 2}} \log\left(\frac{D\,(R_1 + R_2)}{D\,R_1 + R_2}\right)\right] > \log D, \tag{S.104}$$

which reduces to

$$D^x \exp(y) > \frac{DR_1 + R_2}{R_1 + R_2}, \tag{S.105}$$

where

$$\begin{aligned} x &= \frac{g_{\beta 1}\,(g_{\gamma 2} - g_{\beta 2})}{g_{\gamma_2}\,(g_{\beta 1} - g_{\beta 2})} \\ y &= \frac{g_{\beta 1}\,g_{\beta 2}\,\tau_{12}^{(\beta)}}{g_{\beta 1} - g_{\beta 2}} \end{aligned} \tag{S.106}$$

Note that at large enough lag values $\tau_{12}^{(\beta)}$, when the left-hand side of (S.105) is greater than D, Eq. (S.105) is automatically satisfied for all values of R_1/R_2. So, we will always have complementarity if

$$\boxed{\tau_{12}^{(\beta)} > \frac{g_{\beta 1} - g_{\gamma 2}}{g_{\beta 1}\,g_{\gamma 2}} \log D} \tag{S.107}$$

for all B_β with the top choice R_1. If $g_{1\,min} > g_{2\,max}$, we will also have an anomalous microbe among survivors: it will be the expert on R_2 among those whose top choice is also R_2. This is where **the anomalous metabolic strategy** pays off: when the lag is long enough, all the microbe with the top choice R_1 will compete for that particular nutrient, and, among those, there can be only one survivor. In this case, even if a species grows better on R_1, when everyone is in favour of R_1 for that same reason, it might make a wise decision to switch to the top choice R_2.

If however (S.107) does not hold, then (S.105) reduces to

$$\boxed{\frac{R_1}{R_2} < \frac{D^x\,\exp(y) - 1}{D - D^x\,\exp(y)}}, \tag{S.108}$$

with x and y given by (S.106). Substituting $R_1/R_2 = 1$ and assuming $D \gg 1$ we get the condition under which the complementarity is preserved in the case of the **balanced nutrient supply**:

$$\boxed{\tau_{12}^{(\beta)} > \frac{g_{\beta 1} - g_{\gamma 2}}{g_{\beta 1}\,g_{\gamma 2}} \log D - \frac{g_{\beta 1} - g_{\beta 2}}{g_{\beta 1}\,g_{\beta 2}} \log 2} \tag{S.109}$$

K.3 In which way non-similarity of the growth rate distributions on different sources affects diversity

Here we consider, again, an environment with two nutrients available for consumption. If all the growth rates on one nutrient, say, R_1 are much greater than those on another nutrient, say, R_2, the diversity may be out of the question as the lion share of (multiplicative) growth will be done on the most efficient nutrient, the main object of competition between invading microbes. So in this subsection we concentrate on the possibility for an ordinary (non-expert) species with the top choice R_1 to coexist with the expert (the one that grows fastest on R_1) with the same top choice.

Supplementary Figure 29: **Diversity in presence of a "super-nutrient"**. An expert microbe B_α (top choice R_1, magenta ZNGI) does not drive out the microbe B_β (top choice R_1, orange ZNGI) if its one microbe line (dashed magenta line with an arrow) lands on its ZNGI where the rival species B_β can grow. If the opposite is true for any microbe that shares the food preferences with the expert B_α, and if the complementarity is not possible, there can be no diversity.

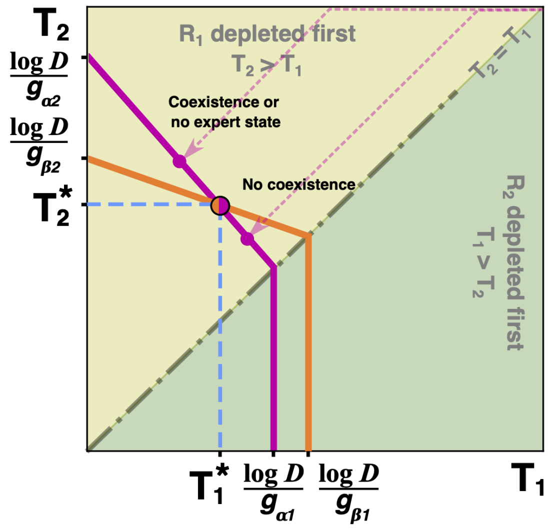

In Fig. 29 the situation favouring coexistence of an expert B_α with another microbe with the same food preferences B_β is presented. The one microbe lines of each of two rival species land on the corresponding ZNGI lines (magenta ZNGI for B_α, orange one for B_α) at the point of their monoculture steady states. B_β can invade the monoculture of B_α if

$$g_{\beta 1}\, T_1^{(\alpha)} + g_{\beta 2}\, T_2^{(\alpha)} > \log D, \tag{S.110}$$

where

$$\begin{aligned} T_1^{(\alpha)} &= \frac{1}{g_{\alpha 1}} \log\left(\frac{D\, R_1 + R_2}{R_1 + R_2}\right) \\ T_2^{(\alpha)} &= T_1^{(\alpha)} + \frac{1}{g_{\alpha 2}} \log\left(\frac{D\,(R_1 + R_2)}{D\, R_1 + R_2}\right), \end{aligned} \tag{S.111}$$

$T_1^{(\alpha)}$, $T_2^{(\alpha)}$ being the depletion times of the B_α−monoculture.

Eq. (S.110) reduces to

$$\boxed{\frac{R_1}{R_2} < \frac{D^h - 1}{D - D^h}}, \tag{S.112}$$

where

$$h = \frac{g_{\alpha 1}\,(g_{\beta 2} - g_{\alpha 2})}{g_{\alpha 1}\, g_{\beta 2} - g_{\alpha 2}\, g_{\beta 2}}. \tag{S.113}$$

In the case of the balanced nutrient supply $R_1 = R_2$ and $D \gg 1$ the condition (S.113) can be reduced to

$$\boxed{\frac{g_{\alpha 2}\,(g_{\alpha 1} - g_{\beta 1})}{g_{\alpha 1}\,g_{\beta 2} - g_{\alpha 2}\,g_{\beta 1}} < \frac{1}{\log_2 D}}. \tag{S.114}$$

If there exists at least one microbe B_β such that the condition (S.114) holds, the diversity can be preserved at the balanced resource supply even for non-similar growth rate distributions.

The regrowth lags as well as the diauxic lags may play their part in preserving or destroying the diversity in an obvious way; in the above, we have neglected their effect on the resulting diversity.

K.4 Multiple resources, one supernutrient

If there is one supernutrient and a lot of other ordinary nutrients (the latter inspiring similar growth rate distributions for the microbes that use them) present in the environment, we can put together the results of two previous subsections to reach a conclusion concerning the fate of both diversity and the complementarity of a steady state community arising as a result of a long series of species invasions. Indeed, if the parameters of the distributions are such that the complementarity can be preserved in a medium containing one supernutrient and any one of the other ordinary nutrients, we can reasonably expect it to be preserved in the given environment with all the multiple resources present as well. The reasons for that conclusion is that, on average, a microbe uses two or three nutrients during a dilution cycle, and almost never the whole set, as those get consumed side by side, and the multiplication provided by the third resource is even less than that provided by the second one. So the prediction based on two nutrients environment should be good enough. If however the parameters are such that there can be no complementarity, but the diversity is preserved, then we will observe the **complementarity with respect to the second choice**. Indeed, if a microbe B_δ with the top choice R_1 and the second choice R_3 can coexist with B_α as well as B_β with the top choice R_1 and the second choice R_2, the niche separation will occur upon depletion of R_1, so that they can both survive.

References

[1] Barthe, M. *et al.* Availability of the molecular switch xylr controls phenotypic heterogeneity and lag duration during escherichia coli adaptation from glucose to xylose. *Mbio* **11**, e02938–20 (2020).

[2] Tilman, D. Resource competition and community structure. *Monographs in population biology* **17**, 1–296 (1982). URL `http://www.ncbi.nlm.nih.gov/pubmed/7162524`.

[3] Tilman, D. Resources: A graphical-mechanistic approach to competition and predation. *Amer Nat* **116**, 362–393 (1980). URL `http://dx.doi.org/10.1086/283633`.

[4] Tilman, D. Resource competition and community structure. *Monographs in population biology* **17**, 1–296 (1982). URL `http://www.ncbi.nlm.nih.gov/pubmed/7162524`.

[5] Goyal, A., Dubinkina, V. & Maslov, S. Multiple stable states in microbial communities explained by the stable marriage problem. *The ISME journal* **12**, 2823–2834 (2018).

[6] Dubinkina, V., Fridman, Y., Pandey, P. P. & Maslov, S. Multistability and regime shifts in microbial communities explained by competition for essential nutrients. *Elife* **8**, e49720 (2019).

[7] Wolf, Y. I., Katsnelson, M. I. & Koonin, E. V. Physical foundations of biological complexity. *Proceedings of the National Academy of Sciences* **115**, E8678–E8687 (2018).

[8] Huisman, J. & Weissing, F. J. Biological conditions for oscillations and chaos generated by multispecies competition. *Ecology* **82**, 2282–2695 (2001).

[9] Basan, M. *et al.* A universal trade-off between growth and lag in fluctuating environments. *Nature* **584**, 470–474 (2020).

[10] Wang, J. *et al.* Natural variation in preparation for nutrient depletion reveals a cost–benefit tradeoff. *PLoS Biol* **13**, e1002041 (2015).

[11] Rolfe, M. D. *et al.* Lag phase is a distinct growth phase that prepares bacteria for exponential growth and involves transient metal accumulation. *Journal of bacteriology* **194**, 686–701 (2012).
